# Somatic FOXC1 insertion mutation remodels the immune microenvironment and promotes the progression of childhood acute lymphoblastic leukemia

**DOI:** 10.1038/s41419-022-04873-y

**Published:** 2022-05-03

**Authors:** Yaping Wang, Xiaopeng Ma, Jie Huang, Xiaoyun Yang, Meiyun Kang, Xiaoyan Sun, Huimin Li, Yijun Wu, Heng Zhang, Yuting Zhu, Yao Xue, Yongjun Fang

**Affiliations:** Department of Hematology and Oncology, Children’s Hospital of Nanjing Medical University, Nanjing Medical University, 72# Guangzhou Road, Nanjing, Jiangsu Province China

**Keywords:** Acute lymphocytic leukaemia, Cancer epigenetics

## Abstract

Acute lymphoblastic leukemia (ALL) is the most common malignant hematological diseases in children. An immunosuppressive microenvironment, particularly regulatory T cell (Treg) infiltration, has been documented to be highly associated with childhood ALL. This present study, based on genetic factors, was aimed at investigating the mutations potentially involved in the immunosuppressive microenvironment in childhood ALL. After whole-exome sequencing was used on DNA extracted from the T cells of ALL bone marrow samples, we found the FOXC1 H446HG induced a increased Treg while decreased cytotoxic T lymphocyte (CTL) in bone marrow. The mutation of FOXC1 in T cell promoted the proliferation of leukemia cells in vitro and in vivo. CpG islands formed by insertion mutation led to an abnormal increase in exon methylation and were associated with the suppression of FOXC1. Decreased FOXC1 attenuated the transcription of HDAC1, thus resulting in the activation of KLF10 through increasing H3K27 acetylation in the promoter region. In conclusion, the de novo insertion mutation in FOXC1 induced suppression of FOXC1, thereby promoting a Treg/CTL shift in the ALL immune microenvironment. The FOXC1 H446HG mutation might be a potential therapeutic target for ALL in the future.

## Introduction

Leukemia is a common hyperplastic disease of the hematopoietic system, this malignant proliferative disease appears to be blocked at a certain stage in the differentiation process of hematopoietic stem cells [1, 2]. Acute leukemia is a major disease endangering the lives and health of children. Acute lymphoblastic leukemia (ALL) is the most common hematologic disease among children, accounting for approximately 75% of leukemia cases, and the proportion of malignant tumors in children under 15 years old is as high as nearly 30% [3, 4]. With the continual improvements in the diagnosis and treatment of ALL in children, and the application of multi-drug combination therapy, supportive therapy and hematopoietic stem cell transplantation, the 5-year disease-free survival rate of ALL in children exceeds 80–90%, thus making it a curable malignant tumor [5, 6]. However, a considerable number of patients with ALL death because of complications and recurrence of chemotherapy [7]. An in-depth study of the pathogenesis of ALL in children would provide a solid theoretical basis for the discovery of new biological markers and new targeted therapeutic sites, as well as a new diagnostic basis for ALL in children.

To date, the etiology of leukemia has not been fully clear, and current studies suggest that chemical factors, radiation factors, genetic factors and immune factors play important roles in the pathogenesis of leukemia [8]. The immune system’s role in the occurrence and development of tumors is highly complex. On the one hand, many immune cells are directly involved in the inflammatory process and play roles in promoting inflammation and oncogenesis [9]. Simultaneously, the immune system has an immune monitoring function in which diseased cells are removed [10]. In addition, tumors can induce an inhibitory immune microenvironment and suppress immune function through a complex signaling network, thus eventually leading to immune escape of tumor cells [11]. Immune dysfunction in children with ALL is an important cause of refractory recurrence and high mortality [12]. For leukemia, it has been reported that intrinsic properties of acute myelocytic leukemia (AML) cells lead to accumulation of Treg and impair CTL activation [13]. In the pathogenesis of ALL, researchers have found that T cell immunity, an effective part of anti-tumor immunity, can eliminate leukemia cells by releasing cytokines and cytotoxic substances [14]. Correspondingly, leukemia cells affect cell differentiation and proliferation through the release of inhibitory cytokines or other mechanisms [15]. Countering the body’s immune function, anomalies in T cell immunity and its control network have important roles in the disease course and outcomes, thus reflecting changes in antitumor immunity and tumor immune escape as a result of the interaction [16]. The pathogenesis of leukemia has been shown to affect the activated receptors that are cleaved at the T cell surface, for example, the receptors PD1, CTLA4 and others are significantly increased [17, 18]. Inflammation and tumors simultaneously greatly increase the conservation of the regulatory T cell (Treg) environment. Treg cells secrete many inhibitory factors (e.g., TGF and IL10) that suppress the immune response [19]. Activated cells inhibit the activation, proliferation and immune effects of T cells (including cytotoxic T cell and T helper cells) in vivo, as well as the immune responses of cells, cell activation and antibody generation. Studies have shown that the proportion of Treg subsets in children with a variety of hematological malignant diseases, including ALL, was significantly higher than that in healthy individuals. Meanwhile, the abnormal distribution of Tregs in children with ALL has been identified to be closely associated with poor prognosis in patients [20]. During the development and progression of leukemia, Tregs inhibit the anti-tumor effects of other IL-2-dependent effector T cells by secreting CD25. In addition, Tregs inhibit the differentiation of B cells through the granzyme A pathway [21]. In immunotherapy for leukemia, dendritic cells cytokine induced killer cell (DC-CIK) loaded with specific leukemia cells significantly decreases the peripheral Treg distribution in patients with ALL [22]. Although the immunosuppressive and tumor-promoting effects of Tregs in the occurrence and development of ALL in children have been gradually clarified by researchers, the effects of the promotion of Treg differentiation on the occurrence of ALL in children, as well as the specific upstream regulatory mechanism, remain unclear.

In our research on the pathogenesis of ALL in children, we have found that genetic and environmental factors have important roles in the development of childhood ALL [23, 24]. We have observed a series of inductions in the environment of childhood ALL as well as the potential risks of specific mutations in single nucleotide polymorphism loci, thus indicating a significant role of genetic factors in the pathogenesis of childhood ALL. In the study of the mechanism of increased differentiation of Tregs during the pathogenesis of ALL in children, researchers have found that the interaction of peripheral T cells CD27-CD70 increases the expression of the anti-apoptotic factor BCL-2, thereby inhibiting the apoptosis of Treg cells [25]. Meanwhile, mutations in genome-specific genes can lead to abnormal T cell differentiation [26]. However, to date, no reports have explored the regulatory mechanism of the abnormal increase in the Treg distribution during the pathogenesis of ALL in children from a genetic perspective. Therefore, on the basis of previous genetic studies, our team aimed to screen for mutations in T cells associated with ALL and abnormal differentiation of Tregs in children by using high-throughput second-generation sequencing technology.

## Results

### Exome sequencing in bone marrow T cells of patients with ALL

**Fig. 1:**
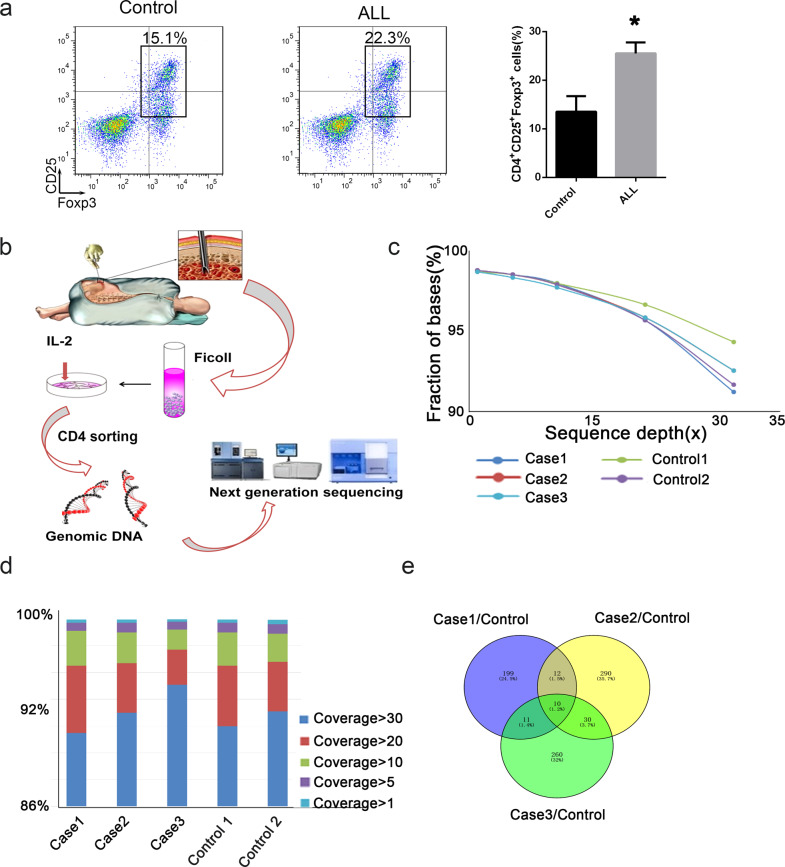
Exome sequencing in the bone marrow T cells of patients with ALL. **a** The percentage of Tregs among T cells was detected by flow cytometry. CD4, CD25 and Foxp3 were used as biomarkers for Tregs. **b** Schematic diagram of genome DNA extraction from immune cells. **c** Sequence depth of exome sequencing. **d** Coverage of exome sequencing. **e** Venny analysis for candidate indels. *p < 0.05.

We first detected the endogenous Treg distribution (CD4^+^CD25^+^Foxp3^+^) in bone marrow samples extracted from patients with ALL and healthy children. As presented in Fig. 1a, an increased Treg percentage was detected in patients with ALL, in agreement with previous results. Next, the CD4^+^ T cells from bone marrow samples were sorted with CD4 magnetic beads. The genomic DNA was extracted after culture with IL-2 for 3 days (Fig. 1b). The sequencing depth and coverage were also confirmed (Fig. 1c, d). The indel mutation was obtained in three patients with ALL and healthy controls. As presented in Fig. 1e, after annotation through multiple databases, we compared the identified indel mutation between the ALL group and the healthy controls. If the newly discovered SNP sites and the same sites in the ALL group were not consistent with the bases in the control group, they were considered candidate difference sites. Finally, we obtained 232 indel mutations in patient 1, 342 mutations in patient 2 and 311 in patient 3. Venny analysis revealed ten common indel mutations in patients with ALL.

### The FOXC1 H446HG mutation is associated with increased Treg distribution in ALL

**Fig. 2:**
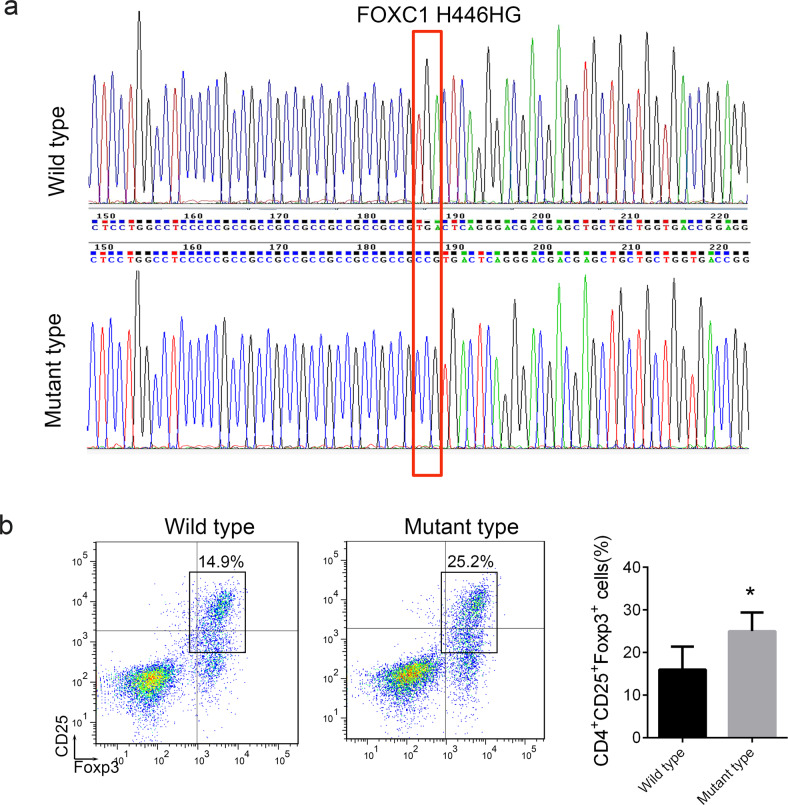
The FOXC1 H446HG mutation is associated with increased Tregs. **a** Sanger sequencing of the FOXC1 H446HG mutation. **b** The percentage of Tregs. *p < 0.05.

**Fig. 3:**
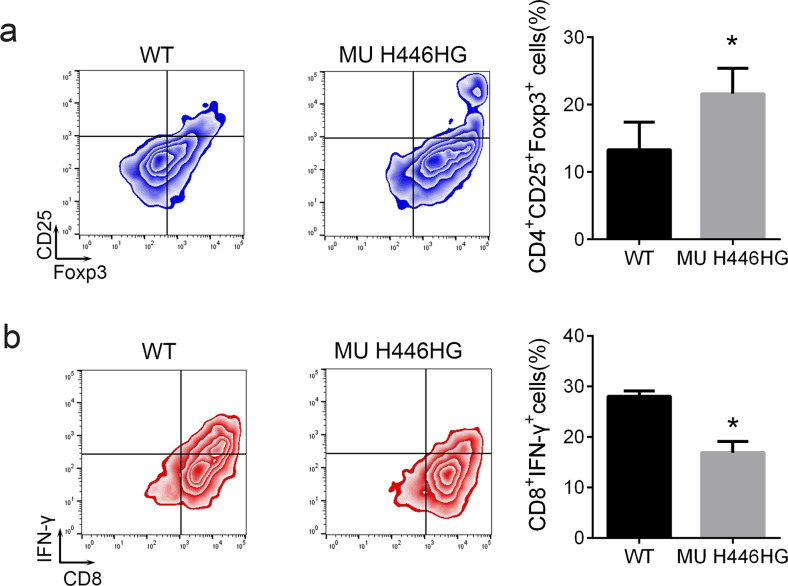
The FOXC1 H446HG mutation induces the Treg/CTL shift. **a** The percentage of Tregs. **b** The percentage of CTL. All experiments were performed in triplicate. *p < 0.05.

The detailed information for candidate indel mutation is presented in Supplementary Table 3. We next performed Sanger sequencing to validate the point, insertion and deletion mutations. We further confirmed that the FOXC1 insertion mutation of changed the 446^th^ amino acid from A to ACGG, thus resulting in the H446HG mutation (Fig. 2a). The remaining mutations were not confirmed by Sanger sequencing. We then further analyzed whether the mutation was associated with the abnormal distribution of Tregs. A total of 20 patients with ALL positive for the H446HG mutation were confirmed by Sanger sequencing out of 500 ALL patients, whereas another 20 ALL children as controls were confirmed not to have the H446HG mutation. The distribution of Tregs was detected by flow cytometry. As presented in Fig. 2b, patients with ALL with the H446HG mutation showed a higher percentage of Tregs. We further constructed a plasmid for expression of mutant FOXC1 through the GeneCopoeia’s IndelCheck™ CRISPR insertion detection system (Supplementary Fig. 1a). The mutant and wild type were confirmed by gel electrophoresis and Sanger sequencing (Supplementary Fig. 1b and c). We generated naïve T cells overexpressing either wild type full length FOXC1 or mutant FOXC1. The distribution of Tregs was further detected. As presented in Fig. 3a, the Tregs increased when mutant FOXC1 was overexpressed. Furthermore, we also found a decreased distribution of cytotoxic T cells (CD8^+^INF-γ^+^) in cells treated with mutant overexpression lentivirus (Fig. 3b). However, no difference was found in the distribution of Th1, Th2 or Th17, thus indicating that the H446HG mutation might have induced a Treg/CTL shift in the immune microenvironment in ALL (Supplementary Fig. 2a–c).

### The FOXC1 H446HG mutation promotes cell proliferation in vitro and in vivo

**Fig. 4:**
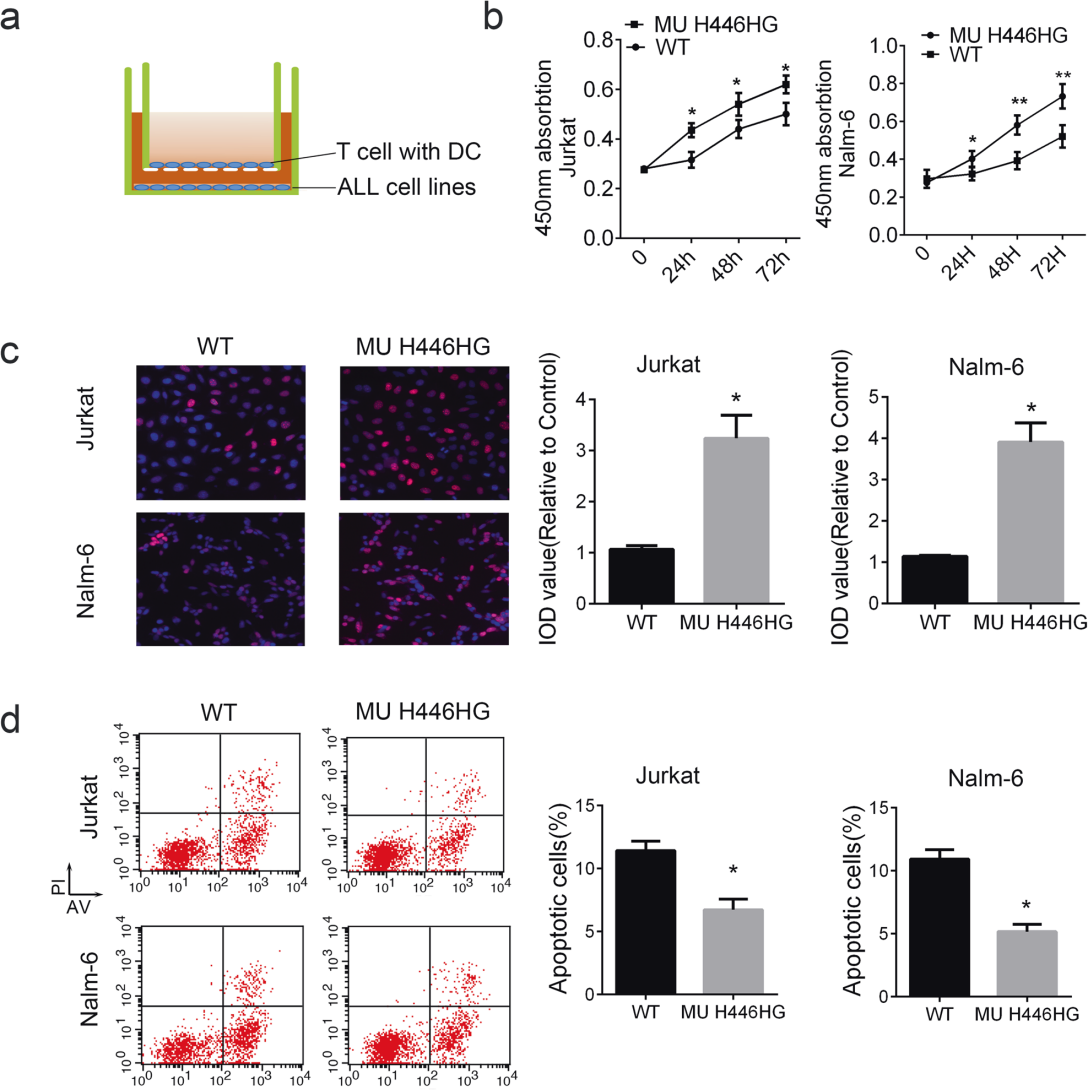
The FOXC1 H446HG mutation promotes cell proliferation. **a** Schematic diagram of the co-culture system. **b** CCK8 assay. **c** EDU assay. **d** Cell apoptosis detected by flow cytometry. All experiments were performed in triplicate. *p < 0.05. **p < 0.01.

**Fig. 5:**
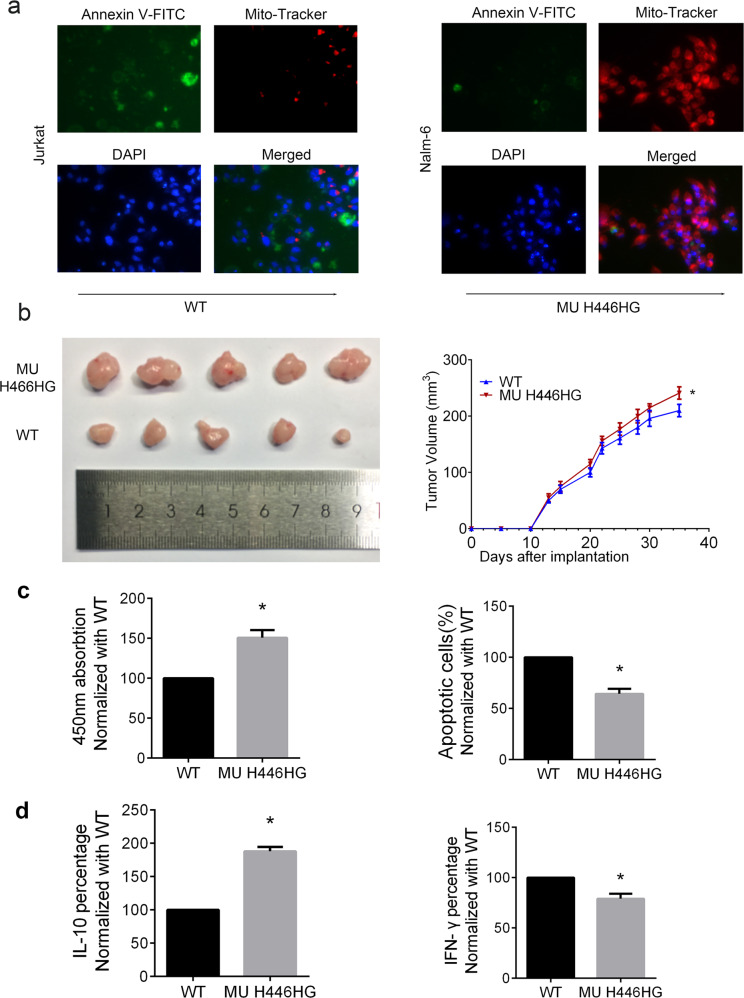
The FOXC1 H446HG mutation decreases cell apoptosis and promotes tumor growth. **a** Mitochondrial membrane potential detection through immunofluorescence with Annexin V-FITC (green), Mito-Tracker Red CMXRos (red) and DAPI (blue). **b** Mice with established tumors in different groups were imaged. Quantification of tumor growth was measured almost every 3 days. **c** Cell proliferation was measured by CCK8. Cell apoptosis were investigated by flow cytometry. **d** Cytokines secreted by T cells were investigated by flow cytometry. Data were normalized with control group (wild group). All experiments were performed in triplicate. *p < 0.05.

We further examined whether the reprogramming caused by the H446HG mutation in the immune microenvironment was associated with tumor cell biological behavior. We first established a co-culture system of immune cells and leukemia cells (both Jurkat as T-ALL type and Nalm-6 as B-T-ALL type). Naïve T cells were treated with FOXC1 wild type lentivirus or H446HG mutant lentivirus. As presented in Fig. 4a, the T cells loaded with DC antigens was seeded in the upper chamber while the leukemia cells were cultured in the lower chamber. After co-culturing, cell proliferation was measured with CCK8 and EDU. Cell proliferation was promoted by co-culturing with T cells treated with H446HG mutant lentivirus (Fig. 4b). The EDU assay also confirmed this result (Fig. 4c). We next detected apoptosis in leukemia cells, as presented in Fig. 4d. Apoptosis was suppressed when the immune cells overexpressed mutant FOXC1, in agreement with the abnormal cell proliferation findings. These results indicating the consistently positive effect of H446HG mutated T cells on tumor cell proliferation in different ALL subtypes. Furthermore, we also investigated cell apoptosis by immunofluorescence detection of the ΔΨm mitochondrial membrane potential. As presented in Fig. 5a, upregulated FOXC1 H446HG decreased apoptosis in leukemia cells. A subcutaneous xenograft model was constructed by subcutaneous injection of leukemia cells co-cultured with immune cells into NOD/SCID mice. Tumor growth was promoted by treatment of immune cells with FOXC1 H446HG overexpressing lentivirus compared with the wild type (Fig. 5b).

Besides, we employed the Jurkat drived xenograft mice model to investigate the cell proliferation, apoptosis and the cytokines secreted by Treg and CTL. As presented in Fig. 5c, cell proliferation was evaluated with the mutation type of FOXC1 while the apoptosis was reduced. IL-10 secreted by Treg was increased while IFN- γ secreted by CTL was decreased in T cells treated with mutant proteins (Fig. 5d).

### The FOXC1 H446HG mutation causes hypermethylation of the FOXC1 exon region

**Fig. 6:**
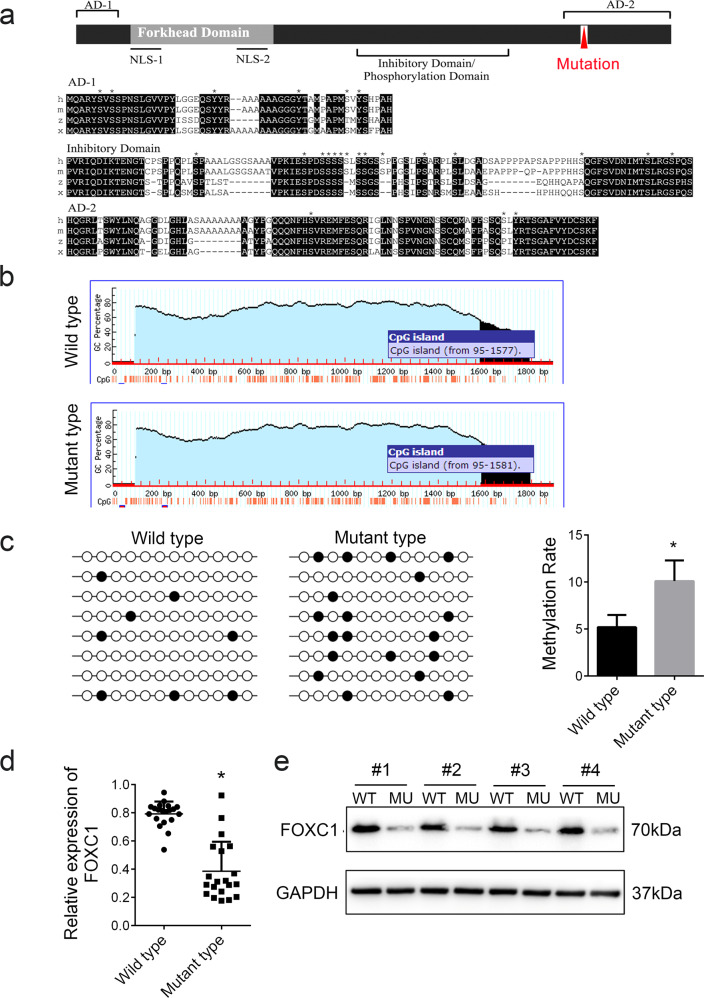
The FOXC1 H446HG mutation is associated with FOXC1 suppression. **a** The location of the mutation in the FOXC1 protein domain. **b** CpG island prediction. **c** The methylation level of FOXC1, detected by bisulfite sequencing. **d** Relative mRNA expression of FOXC1. **e** Protein level of FOXC1. All experiments were performed in triplicate. *p < 0.05.

To shed light on the detailed mechanism underlying how the H446HG mutation induced the Treg/CTL shift, we next analyzed the detailed position of the H446HG mutation through FOXC1 domain analysis. The H446HG mutation was found to be located in the trans-activation domain 2(AD-2) of FOXC1 (Fig. 6a). Previous studies have shown that the deletion of residues in the 435–475 region significantly inhibits the expression of FOXC1 [27], thus suggesting that this mutation may be associated with the expression of FOXC1, particularly at the transcriptional level. Because the inserted nucleotide sequence had a high abundance of GC, we next analyzed whether the CpG island might be altered by the H446HG mutation. As predicted, the CpG island was extended from 1577 to 1581 after the H446HG insertion mutation (Fig. 6b). Further bisulfite sequencing revealed that the H446HG mutation induced the methylation of the exon region (Fig. 6c). RT-PCR also confirmed that the hypermethylation of FOXC1 decreased the expression of FOXC1 (Fig. 6d). We also detected the expression of FOXC1 protein levels in the T cells of patients with ALL; interestingly, we confirmed the decreased FOXC1 levels in patients with the mutation (Fig. 6e).

### Decreased FOXC1 promotes leukemia cell proliferation through inducing a Treg/CTL shift

**Fig. 7:**
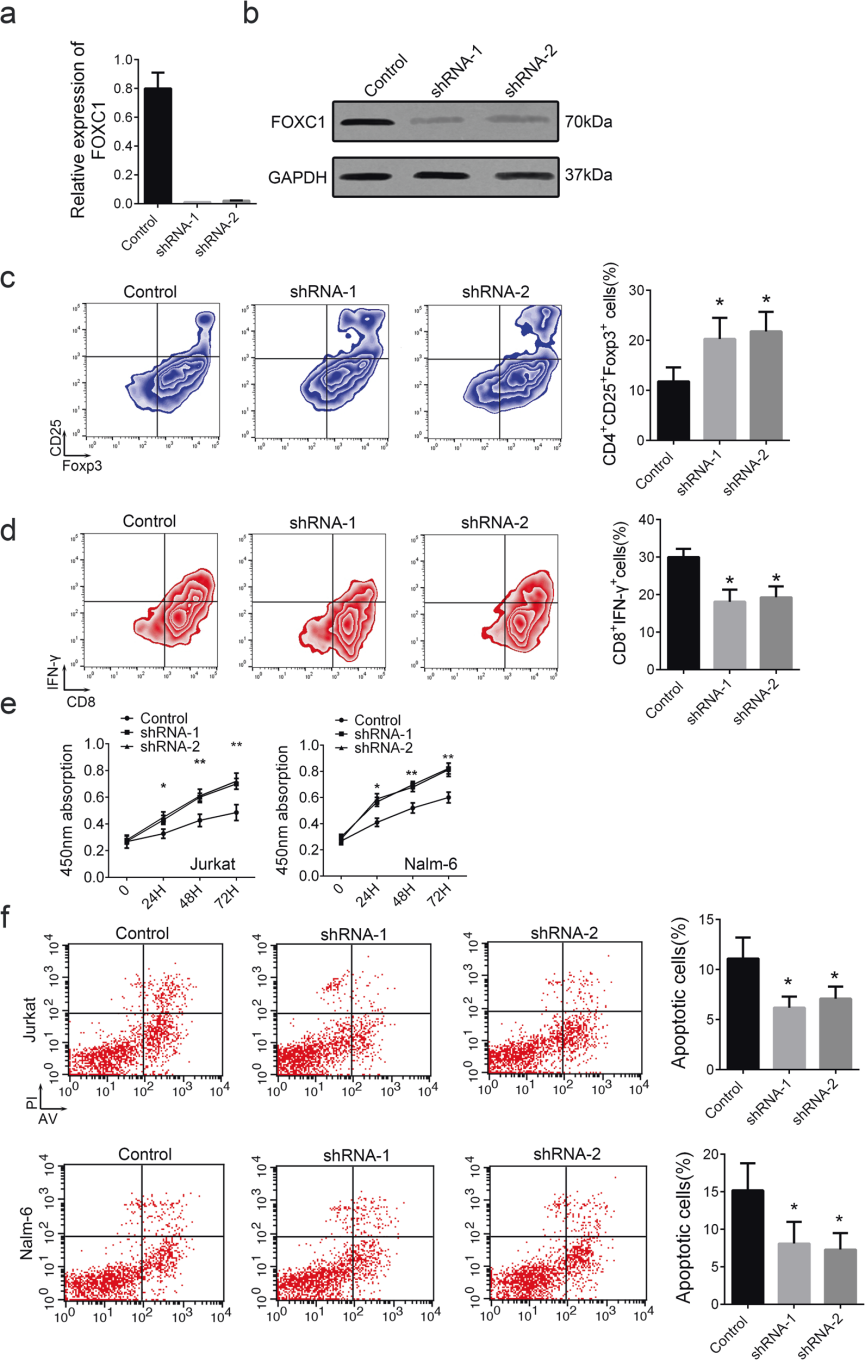
Decreased FOXC1 in T cells promotes leukemia cell proliferation. **a** Relative expression of FOXC1 treated with shRNAs. **b** Relative protein level of FOXC1 treated with shRNAs. **c** Treg distribution. **d** CTL distribution. **e** CCK8 assay, **f** Cell apoptosis detected by flow cytometry. All experiments were performed in triplicate. *p < 0.05. **p < 0.01.

Because the H446HG mutation caused hypermethylation of FOXC1 and suppressed FOXC1 expression, we next used shRNA technology to knock down FOXC1. The knockdown efficiency for two independent shRNA targeting sites was confirmed by RT-PCR and western blotting (Fig. 7a, b). The increased distribution of Tregs was confirmed with FOXC1 suppression, whereas the CTL percentage was promoted (Fig. 7c, d). We also investigated the function of the Treg/CTL shift on leukemia cells through a co-culture system (Fig. 7e). The proliferation of leukemia cells was elevated in the mutant group. Furthermore, flow cytometry confirmed a decrease in apoptotic cells in the presence of the H446HG mutant (Fig. 7f).

**Fig. 8:**
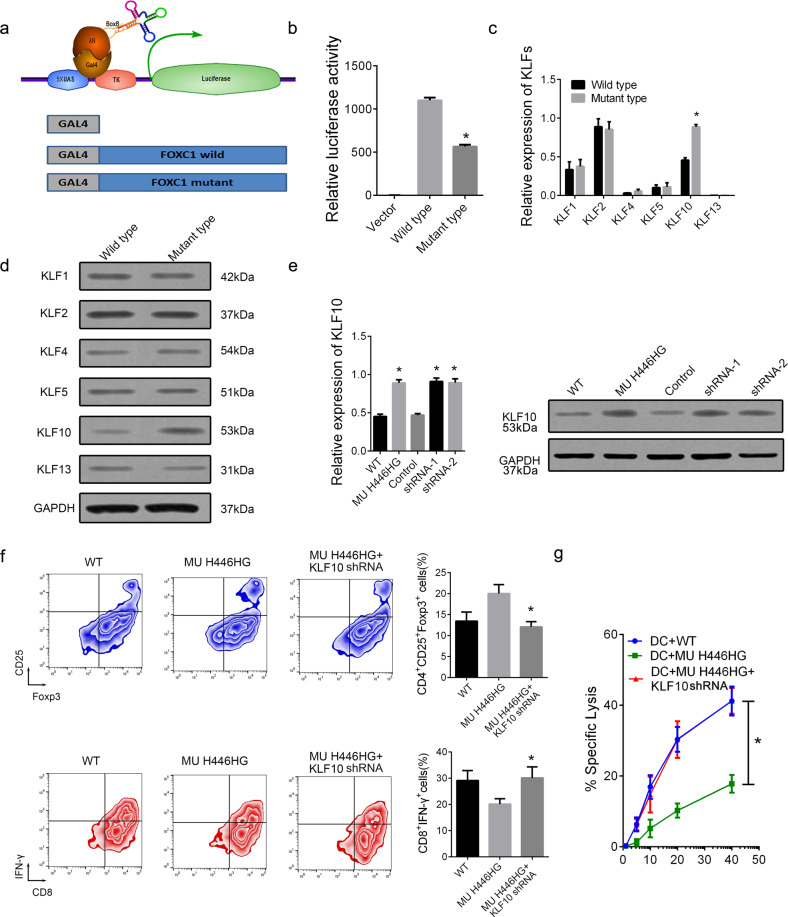
Decreased FOXC1 promotes KLF10. **a** Schematic diagram of the Gal4-λn/Boxb reporter gene system. **b** Relative luciferase activity of FOXC1. **c** Relative mRNA expression level of KLFs. **d** Relative protein expression level of KLFs. **e** Relative expression level of KLF10 in T cells treated with mutant or wild type FOXC1 and shRNAs targeting FOXC1. **f** Treg and CTL distribution. **g** The cytolytic activity of the generated CTLs co-cultured with T cells with different treatments, detected by ^51^Cr-release assays. All experiments were performed in triplicate. *p < 0.05.

We also used the Gal4-λn/BoxB reporter gene system [28] to fuse FOXC1 wild-type and mutant to BoxB sequences and to determine the effect of this mutation on the FOXC1 expression level in naïve T cells by detecting the luciferase activity at the tail end (Fig. 8a). The FOXC1 H446HG mutation inhibited FOXC1 expression, showing lower luciferase activity than that of wild-type FOXC1 (Fig. 8b).

### Mutant FOXC1 increases KLF10 through epigenetic modification

In studies of the downstream regulatory mechanisms of FOXC1, researchers have found abnormally expressed KLFs in FOXC1 knockout mice. In patients with acute myeloid leukemia (AML), ectopic expression of FOXC1 significantly inhibits KLF4 expression. The KLF family plays an important role in the hematopoietic system and in the acquired immune microenvironment mediated by T cells and B cells [29]. However, the mechanisms involved in T cell differentiation and the occurrence and development of childhood ALL remain unclear. To further clarify whether the low expression of FOXC1 mediated by the FOXC1 H446HG mutation causes abnormal differentiation of Tregs through the KLFs family dependent signaling pathway, we further detected the expression of KLFs (KLF1, 2, 4, 5, 10 and 13) in the T cells of FOXC1 mutant patients with ALL and healthy controls. The expression of KLF10 significantly increased in the bone marrow T cells of patients with ALL carrying the FOXC1 mutation (*p* = 0.02), whereas there was no significant difference in the expression of the rest KLF family members including KLF1, 2, 4, 5and 13 (*p* = 0.12, Fig. 8c, d). Notably, KLF10 plays an important role in Treg differentiation. For example, in TGF-β-induced Treg directed differentiation, KLF10 activates TGF-β1 and Foxp3 at the transcriptional level, thus inducing the production of Tregs, whereas in T-cell-specific KLF^−/−^^T-CKO^ mice, TGF-β-directed Treg induction was significantly inhibited. The increased level of KLF10 was also confirmed in T cells treated with FOXC1 shRNAs (Fig. 8e). We also observed that KLF10 knockdown in H446HG mutant T cells attenuated the Treg/CTL shift induced by the insertion mutation (Fig. 8f). Furthermore, we performed ^51^Cr-release assays to investigate the suppressive effects on T cells. Jurkat cells were labeled with ^51^Cr, and this was followed by co-culture with Jurkat-vaccinated dendritic cells and T cells. Decreased absorbance was observed in the group co-cultured with H446HG mutant T cells, thus indicating stronger immunosuppressive activity by the H446HG mutant than the wild type (Fig. 8g). Additionally, suppression of KLF10 rescued immune suppressive activity, thus suggesting that H446HG mutation promoted an immunosuppressive microenvironment, on the basis of the presence of KLF10.

**Fig. 9:**
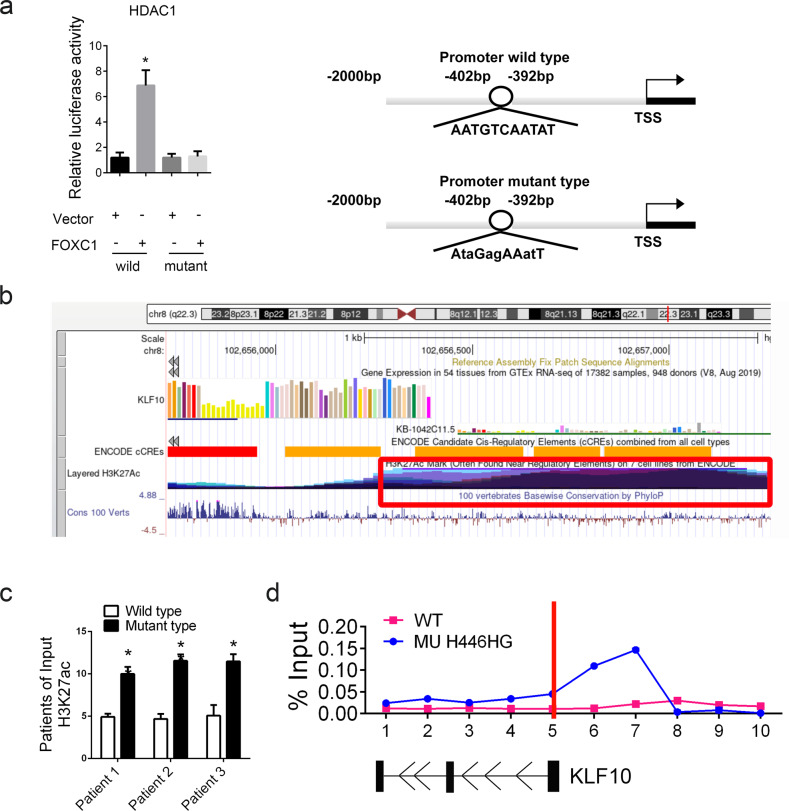
Mutant FOXC1 increases KLF10 through epigenetic modification. **a** Dual-luciferase reporter system detecting the transcription activation of FOXC1 on HDAC1. **b** H3K27ac distribution in the promoter region of KLF10. **c** Enrichment of H3K27Ac in the promoter region of KLF10 in patients with ALL. **d** Enrichment of H3K27Ac, detected through multisite ChIP-PCR around KLF10 loci in cells treated with FOXC1 with different types, as normalized to the input. All experiments were performed in triplicate. Data are presented as means ± SEM. (*p < 0.05).

We further explored the detailed mechanism of how mutant FOXC1 increased the level of KLF10. Based on the intracellular localization of FOXC1, we assumed the FOXC1 might transcriptionally regulate KLF10. FOXC1 was identified as a special transcription factor in cellular biological processes. We next predicted the potential intracellular target of FOXC1. Bioinformatics analysis revealed that HDAC1 might be transcriptionally regulated by FOXC1. The gene-regulation software (http://gene-regulation.com/) was employed for predicting the transcriptional target for FOXC1. We next used the dual-luciferase reporter system to measure the transcriptional activation. We found that mutant FOXC1, compared with the wild type, suppressed the transcription of HDAC1 (Fig. 9a). HDAC1 is an enzyme that removes acetyl groups from the lysine residues of histones and non-histone proteins, thus resulting in the abnormal expression of target genes. We next analyzed the promoter region of KLF10, as presented in Fig. 9b, and identified a high abundance of H3K27ac in the promoter region of KLF10, according to predictions from the ENCODE database. We reasoned that this critical point of regulation might affect the expression of KLF10. We next detected the enrichment in H3K27ac in patients with ALL by using ChIP assays with specific antibodies. We found enhanced acetylation of H3K27 in the promoter of KLF10 in patients with the H446HG mutation. We next analyzed the histone modifications by multisite ChIP-PCR around KLF10 loci in T cells treated with either wild type or mutant FOXC1 overexpression lentivirus. We found that H3K27 acetylation was markedly enhanced around the transcription start site of KLF10 (Fig. 9c), thus indicating that mutant FOXC1 might promote the expression of KLF10 through enhancing H3K27 acetylation via the suppression of HDAC1.

## Discussion

To date, the etiology of leukemia has not been completely clear. The current study revealed that chemical factors, radiation factors, genetic factors and immune factors play important roles in the pathogenesis of leukemia [30]. According to the recent report “Hallmarks of Cancers”, one of the main mechanisms involves active cancer cells’ evasion of attack and elimination was immune cells; this capability highlights the dual roles of an immune system that both antagonizes and enhances tumor development and progression [31]. The immune system in oncogenesis is complicated: many immune cells are directly involved in the inflammatory process and promote the function of inflammation and cancer. Simultaneously, the immune system exerts an immune monitoring function to remove diseased cells and induce a tumor inhibitory immune microenvironment, through a network suppressing the immune complex signaling function, thus eventually leading to the immune escape of tumor cells [32]. In particular, inflammation is the initial stage of neoplastic progression and has been found to foster the development of incipient neoplasias into full-blown cancers [33, 34]. Additionally, inflammatory cells release chemicals, notably reactive oxygen species, that are actively mutagenic to nearby cancer cells, thereby accelerating their genetic evolution toward states of heightened malignancy.

Patients with acute leukemia often have immunodeficiency or deficiency. Previous studies have found abnormal distributions of CD4^+^CD25^+^ regulatory T cells in the peripheral blood of leukemia patients, thus forming a suppressive immune microenvironment [35]. In response, another type of immune cells labelled with CD8^+^ T cells have been implicated. According to the different cytokines secreted by CD8^+^ T cells, also known as cytotoxic T cells (Tc), these cells can be divided into two subsets: Tc1 and Tc2. Tc1 cells produce IL-2, IFN-γ, TNF-β and other cytokines, which mainly mediate the cytotoxic activity of cells. Tc2 cells mainly produce IL-4, IL-5, IL-10 and other cytokines, and assist B cells [36]. Thus, the tumor target cell process is divided into effective target cells, fatal blow and target cell cracking three phases. CD8^+^ T cells exert the main antitumor effect, by first identifying the tumor cell surface through type I tumor antigens presented to the MHC molecules and subsequently targeting cells [37].

As an important member of the forkhead box Fox family, FOXC1 plays important roles in human congenital and tumor diseases. FOXC1 is associated with the TGF-β signaling pathway and inhibits cell proliferation [38]. Furthermore, FOXC1 overexpression affects cell cycle progression, thus indicating that FOXC1 is involved in cell proliferation and tumorigenesis [39]. However, during leukemia, the abnormally high expression of FOXC1 in AML and the interaction between HOXA5/HOXA9, members of the Hox family, can lead to abnormal myeloid differentiation. In a recent study on the myeloimmune microenvironment of AML, researchers have found that FOXC1 expression is significantly inhibited in AML marrow neutrophils and monocytes [40], but its expression in lymphocytes, particularly T cells, has not been reported yet.

We also found that the deletion of residues in the 435–475 region had a significant inhibitory effect on the expression of FOXC1, thus suggesting that this mutation may be associated with the expression of FOXC1, particularly at the level of transcription. In addition, FOXC1 H446HG mutation is caused by abnormal insertion of the CGG base at 1338 in the FOXC1 exon region, together with the GC-rich specificity of its upper and lower sequences, and the abnormal methylation of FOXC1 in human diseases. In patients with AML, ectopic expression of FOXC1 significantly inhibits KLF4 expression [41]. The KLF family plays an important role in the hematopoietic system and in the acquired immune microenvironment mediated by T cells and B cells [42]. However, the mechanisms involved in T cell differentiation and the occurrence and development of childhood ALL remain unclear. Whether the low expression of FOXC1 mediated by the FOXC1 H446HG mutation causes abnormal Treg differentiation through the KLFs family dependent signaling pathway requires further clarification. In TGF-β-induced Treg directed differentiation, KLF10 activates TGF-β1 and Foxp3 at the transcriptional level, thus inducing the production of Tregs, whereas in T-cell-specific KLF^−/− T-CKO^ mice, TGF-β-directed Treg induction is significantly inhibited [43].

In conclusion, here we demonstrated that the novel H446HG insertion mutation in FOXC1 in pediatric ALL is strongly associated with the occurrence of ALL. Furthermore, functional experiments revealed that FOXC1 H446HG induces a Treg/CTL shift generating a suppressive immune microenvironment and finally promoting the development of ALL. We also confirmed that this mutation decreases FOXC1 levels through hypermethylation modifications and attenuates the transcription of HADC1. The increased acetylation of H3K27 in the KLF10 promoter, which is bound by FOXC1, causes abnormal accumulation of KLF10. This study explored the microenvironment of the pathogenesis of pediatric ALL through a genetic viewpoint. Besides, whether regulatory mechanism in other leukemia types such as AML was relevant in ALL need to be further investigated in the future. Our findings may provide a new basis for future interventions involving targeted mutations to alleviate the immunosuppressive microenvironment. This new identified FOXC1 H446HG mutation might provide a basis for future targeted immunotherapy of ALL.

## Materials and methods

### Clinical samples

Bone marrow samples were obtained from pediatric patients newly diagnosed with ALL who received therapy at Children’s Hospital of Nanjing Medical University (Nanjing, China) between 2015 and 2020. We excluded individuals with concurrent autoimmune disease, HIV or syphilis; patients who received immunosuppressive therapy for at least one month; and patients diagnosed with immunodeficiency disease. Clinical characteristics were classified and diagnosed according to the guidelines of the Morphologic, Immunologic, Cytogenetic and Molecular biologic classification technique (MICM). Healthy control samples were obtained from healthy volunteers or children who received bone marrow biopsy tests and were not diagnosed with hematological system diseases. Total 20 ALL clinical samples with mutation (age range from 19 to 82 months, with 10 male and 10 female) were enrolled while another cohort of 20 ALL children without mutation as controls were labelled as control group (age range from 18 to 81 months, with 9 male and 11 female). The All research was performed in compliance with governmental policies and the Declaration of Helsinki and approved by the ethics committee of Children’s Hospital of Nanjing Medical University (2016-SRFA-023). Experiments were undertaken with the understanding of, and written consent.

### Exome sequencing

The DNA concentration was measured with a Qubit® DNA Assay Kit and a Qubit® 3.0 Flurometer (Invitrogen, USA) after extraction. The whole exome library was prepared with an Agilent SureSelect Human All Exon V6 kit (Agilent Technologies Inc.) according to the manufacturer’s standard protocol. Briefly, qualified genomic DNA was fragmented to an average size of 200 bp. The ends of the DNA fragments were subjected to an end repair process, followed by A-tailing and ligation of adapters. DNA fragments with ligated adapters on both ends were selectively enriched by PCR. After PCR, library hybridization and exome capture were performed with a biotin labeled probe and magnetic bead selection. Captured libraries were enriched and tagged by PCR to prepare for sequencing. The final libraries were quantified with a KAPA Library Quantification kit (KAPA Biosystems, South Africa) and an Agilent 2100 Bioanalyzer. Paired-end sequencing (2 × 150 bp) was performed on an Illumina NovaSeq 6000 sequencer (Illumina, CA, USA).

### Quantitative real-time PCR

Total RNAs of cell samples were extracted with TRIzol reagent according to the manufacturer’s instructions (IInvitrogen, Carlsbad, CA, USA). The qRT-PCR was conducted to evaluate the expression level of mRNAs of all relevant genes. To extract RNAs from cytoplasma and cytonucleus, we used the SurePrep Nuclear or Cytoplasmic RNA Purification Kit of Thermo Fisher Scientific (Rochester, Waltham, MA, USA). GAPDH were used as the internal control. The samples were digested with DNase before RT-PCR detection. The detailed primer and sequence information was presented in Supplementary Tables 1 and 2.

### Cell culture

Dissociated cells were filtered through a 150 mm mesh and separated by Ficoll centrifugation, and the mononuclear cells were washed and resuspended in RPMI 1640 supplemented with 10% fetal bovine serum (FBS) (Gibco, CA, USA). Anti-CD4 magnetic Dynalbeads (Invitrogen Life Technologies, CA, USA) were used to purify CD4 (+) T cells for further investigation. IL-2 (PeproTech Inc., Rocky Hill, New Jersey, USA) was appended for the expansion of T cell in vitro.

### Cell proliferation investigation

Cells transfected for 24 h with miRNA mimic or stably transduced cells were seeded into 96-well plates at a density of 1000 cells per well in triplicate. The cells were harvested, and 10 μl of CCK-8 reagent (Dojindo, Kumamoto, Japan) was added to 100 μl of culture medium. The cells were subsequently incubated for 2 h at 37 °C, and the optical density was measured at 450 nm with a microplate reader (SpectraMax i3, Molecular Devices, USA).

We also used an EDU assay kit (C0071S; Beyotime Institute of Biotechnology, Shanghai, China) to measure cell proliferation. Briefly, cells were seeded (5 × 10³ cells/well) into 96-well plates and cultured for 48 h. Subsequently, EDU (100 µl; 10 µM) was incubated with the cells for 3 h at 37 °C. The cells were then fixed in 4% paraformaldehyde for 15 min, permeabilized at room temperature with 0.3% Triton X-100 for 10 min and then washed with 3% BSA. DAPI (100 µl) was added for 10 min to visualize the nuclei. A Nikon TI-DH light microscope (Nikon Corporation, Tokyo, Japan) was used to capture images of the cells. The mean numbers of cells in three fields for each sample were calculated to assess cell proliferation.

### Mitochondrial membrane potential detection

Mitochondrial membrane potential detection was conducted with a Mitochondrial Membrane Potential and Apoptosis Detection Kit with Mito-Tracker Red CMXRos and Annexin V-FITC according to the manufacturer’s instructions (MKBio, Shanghai, China). In brief, after adherent cells were washed with PBS, and Annexin V-FITC and Mito-Tracker Red CMXRos staining were then performed. DAPI was added after 30 min of incubation in the dark for nucleus staining. Fluorescence intensity was observed under a fluorescence microscope.

### In vivo model

Jurkat cells (1 × 10^6^ cells) in 100 μl of buffered saline were subcutaneously injected into the dorsal tissue of 5- to 6-week-old male NOD/SCID mice. The Jurkat cells had been authenticated according to STR profiles and tested for mycoplasma contamination. T cells (2 × 10^6^) overexpressing wild type and mutant proteins were polarized with tumor monocytes (2 × 10^6^) in 100 μl of buffered saline and subsequently injected into the inguen of the male mice on day 3 after cell inoculation. Mock vector was used in the control group. Tumors were measured every week after implantation, and the volume of each tumor was calculated (length × width^2^ × 0.5). All mice were sacrificed 5 weeks afterwards for further analysis. Besides, T cells (2 × 10^6^) overexpressing wild type and mutant proteins were polarized with tumor monocytes (2 × 10^6^) in 100 μl of buffered saline and subsequently injected through the tail vein of the male mice on day 3 after Jurkat inoculation. The T cells and monocytes were injected each a week. All mice were sacrificed after four times consecutive injections. The blood and tumor were collected for further analysis. T cell were enriched by CD3 beads. Cell proliferation was measured by CCK8. Cell apoptosis and cytokines secreted by T cells were investigated by flow cytometry. The investigators were blinded to the group allocation during the experiment.

### Methylation detection

Genomic DNA was isolated from cells with a Genomic DNA Purification Kit (Promega, USA), then treated with sodium bisulfite (QIAGEN, USA). Primers for amplifying the FOXC1 exome region bisulfite-modified regions are listed in Supplementary Table 1.

### Gal4-λN/BoxB reporter assays

In this system, the BoxB RNA stem loop was fused as described previously [38]. Plasmids encoding a TK-luciferase gene under the control of five GAL4 UAS sites were co-transfected with plasmids encoding GAL4-λN peptide fused to a C-terminal GFP tag and BoxB, as described above. Renilla luciferase was used as a control in this system. The binding of the Gal4-λN fusion was confirmed first.

### Flow cytometry

T cells extracted from bone marrow from children with T-ALL or healthy controls were stained with fluorochrome-conjugated antibodies and then analyzed by flow cytometry. In brief, the T cells were stimulated with leukocyte activation cocktail (BD Pharmingen, CA, USA) at 37 °C for 5 h. Different antibodies for detecting T cell markers were added, incubated and then finally used to stain intracellular markers. Data were acquired on a BD FACSVerse™ flow cytometer (BD Pharmingen, CA, USA). Cell apoptosis were detected by Annexin V/ANXA5-FITC Apoptosis Detection Kit (ab14082, Abcam, CA, USA). The detailed antibodies information was presented in Supplementary Table 4

### Luciferase reporter assays

Cells at a density of 2 × 10^5^ cells per well were incubated in a 24-well plate. The wild type FOXC1 firefly luciferase reporter was transfected with Lipofectamine 2000. pRL-TK was used as a normalization control. The mutated (MT) FOXC1 firefly luciferase reporter was generated with a site directed mutagenesis kit (KM131204, Tiangen, China) according to the manufacturer’s instructions. The mutated vector was used as a negative control. After 48 h, luciferase substrates for firefly and Renilla luciferase were added to the cell lysate in each group. The Dual-Glo Luciferase Assay System (GM-040501A, Genomeditech) was used for measuring luciferase activity with an Infinite 1000 instrument.

### Standard ^51^Cr-release assays (CTL assays)

Cells were transfected with RNA and labeled with ^51^Cr sodium chromate in X-VIVO 20 medium for 1 h at 37 °C. Target cells (1 × 10^4^) were transferred to the wells of a round-bottomed 96-well plate. Various numbers of CTLs were added to a final volume of 200 μL and incubated for 4 h. At the end of the assay, supernatants (50 μL per well) were harvested. The percentage of specific lysis was calculated with the formula (experimental release–spontaneous release)/(maximal release–spontaneous release) × 100%. Spontaneous and maximal release was determined in the presence of X-VIVO 20 medium or 2% Triton X-100, respectively.

### Chromatin immunoprecipitation

ChIP was performed by using the ChIP assay kit according to the manufacturer’s protocol (17-610; Millipore). Formaldehyde was used to crosslink the proteins to the DNA for 20–30 min. Then sonicate lysate to shear DNA to a fragment size of 200–1000 bp. After determination of DNA concentration and fragment size, we add the primary antibody, anti-H3K27m3, anti-H3K27ac, anti-H3K4m3 and IgG, and protein A/G beads into the samples and incubated overnight at 4 °C. The crosslinking was reversed by incubation at 65 °C for 4 h. The DNA was recovered by phenol/chloroform extraction. The primers were used to detect the promoter region by PCR.

### Statistical analysis

Data are presented as mean ± SEM with no special instructions. χ^2^ tests, Student’s t-test and analysis of variance were used to evaluate statistical differences in demographic and clinical characteristics. Pearson correlation analysis was used to analyze the relationships among associated factors. Statistical analysis was performed in STATA 10.0, and the results were graphed with GraphPad Prism software (CA, USA). In all cases, *P* < 0.05 was considered significant.

## Supplementary information

Authorship agreement

aj-checklist

SUPPLEMENTAL MATERIAL

## Data Availability

Microarray data are available in the SRA database (https://www.ncbi.nlm.nih.gov/sra/) under accession number https://www.ncbi.nlm.nih.gov/sra/PRJNA739399.
