# Supplementary material for: Somatic FOXC1 insertion mutation remodels the immune microenvironment and promotes the progression of childhood acute lymphoblastic leukemia
Source: Cell Death Dis. 2022 May 3;13(5):431. doi: 10.1038/s41419-022-04873-y (PMC9065155; doi:10.1038/s41419-022-04873-y)
Supplement: Supplementary file 1 — Authorship agreement [file 41419_2022_4873_MOESM1_ESM.pdf]

<< 返回 | 回复 | 回复全部▾ | 转发 | 删除 | 举报 | 标记为▾ | 移至▾ | 更多▾

Re: Authorship

发件人： jnjmu2021 <jnjmu2021@163.com>

时 间： 2022年04月18日 09:38 星期一 展示更多

Confirmed and Agree
Jie Huang

jnjmu2021

jnjmu2021@163.com

签名由 网易邮箱大师 定制

On 4/18/2022 09:33， wrote：

Dear my colleagues,

Here we need to make an approvement regarding the authorship of manuscript entitled "Somatic FOXC1 insertion mutation remodels the immune microenvironment and promotes the progression of childhood acute lymphoblastic leukemia" which has been principle accepted by Cell Death & Disease (CDDIS-21-4509RRR). In detailed, Jie Huang was added as the co-first author of the article (ranking third). You, as a co-author of this article, agree to the above modifications and confirm that there is no conflict of interest. Please response whether you agree or not.

Thank you again for your work.

Best regards,

Yongjun fang M.D. Ph.D

Department of Hematology/Oncology

the Affiliated Children\'s Hospital of Nanjing Medical University

72 Guangzhou Rd，Nanjing,China

210008，+8618951769586

<< 返回 | 回复 | 回复全部 | 转发 | 删除 | 举报 | 标记为 | 移至 | 更多

Re: Authorship

发件人：王娅萍 <15050564516@163.com>

时　间：2022年04月18日 09:37 星期一 展示更多

I agree with the authorship.
Yaping Wang

王娅萍

wyp_0919@163.com

签名由 网易邮箱大师 定制

On 4/18/2022 09:33， wrote：

Dear my colleagues,

Here we need to make an approvement regarding the authorship of manuscript entitled "Somatic FOXC1 insertion mutation remodels the immune microenvironment and promotes the progression of childhood acute lymphoblastic leukemia" which has been principle accepted by Cell Death & Disease (CDDIS-21-4509RRR). In detailed, Jie Huang was added as the co-first author of the article (ranking third). You, as a co-author of this article, agree to the above modifications and confirm that there is no conflict of interest. Please response whether you agree or not.

Thank you again for your work.

Best regards,

Yongjun fang M.D. Ph.D

Department of Hematology/Oncology

the Affiliated Children\'s Hospital of Nanjing Medical University

189邮箱

VIP fyj322@189.cn 升级VIP服务 手机版 设置 帮助 退出

首页 联系人 附件中心 我的日程 收件箱 云会议 Re:Auth... Re:Auth... Re:Auth... Re:Auth... Re:Auth... Re:Auth...

邮件搜索

写邮件

收件箱(4394)
星标邮件
已发送
草稿箱
垃圾箱
发票夹
微信文件
我的账单 (5)
其他文件夹 (694)
自定义标签
其他邮箱 (650)

返回 回复 回复全部 转发 删除 举报 标记为 移至 更多

Re: Authorship

发件人: Yutting Zhu <maomaozhu1993@163.com>

时 间: 2022年04月18日 11:11 星期一 展示更多

Agree+Yuting Zhu

---- Replied Message ----

From	fyj322
Date	04/18/2022 09:33
To	王娅萍, maxiaopeng@njmu.edu.cn, 15895323665@163.com<15895323665@163.com>, yunmeik_28@126.com, njchsxy@163.com, lhm13813743029@163.com, wyj15380168896@163.com, zhangheng0513@126.com, maomaozhu1993@163.com, yaoyao82986@126.com, jnjmu2021@163.com
Subject	Authorship

Dear my colleagues,

Here we need to make an approvement regarding the authorship of manuscript entitled "Somatic FOXC1 insertion mutation remodels the immune microenvironment and promotes the progression of childhood acute lymphoblastic leukemia" which has been principle accepted by Cell Death & Disease (CDDIS-21-4509RRR). In detailed, Jie Huang was added as the co-first author of the article (ranking third). You, as a co-author of this article, agree to the above modifications and confirm that there is no conflict of interest. Please response whether you agree or not.

Thank you again for your work.

Best regards,

Yongjun fang M.D. Ph.D

Department of Hematology/Oncology

the Affiliated Children\'s Hospital of Nanjing Medical University

72 Guangzhou Rd，Nanjing,China

210008，+8618951769586

189邮箱 VIP fyj322@189.cn 升级VIP服务 手机版 设置 帮助 退出
首页 联系人 附件中心 我的日程 收件箱 云会议 Re:Auth... Re:Auth... Re:Auth... Re:Auth... Re:Auth... Re:Auth... Re:Auth... 邮件搜索
写邮件
收件箱(4393)
星标邮件
已发送
草稿箱
垃圾箱
发票夹
微信文件
我的账单 (5)
其他文件夹 (694)
自定义标签
其他邮箱 (650)
返回 回复 回复全部 转发 删除 举报 标记为 移至 更多

Re: Authorship

发件人：南京市儿童医院医务人员 <lhm13813743029@163.com>

时 间：2022年04月18日 12:45 星期一 展示更多

Agree. Huimin li

lhm13813743029

邮箱：lhm13813743029@163.com

签名由 网易邮箱大师 定制

On 04/18/2022 09:33, fyj322@189.cn wrote:

Dear my colleagues,

Here we need to make an approvement regarding the authorship of manuscript entitled “Somatic FOXC1 insertion mutation remodels the immune microenvironment and promotes the progression of childhood acute lymphoblastic leukemia” which has been principle accepted by Cell Death & Disease (CDDIS-21-4509RRR). In detailed, Jie Huang was added as the co-first author of the article (ranking third). You, as a co-author of this article, agree to the above modifications and confirm that there is no conflict of interest. Please response whether you agree or not.

Thank you again for your work.

Best regards,

Yongjun fang M.D. Ph.D

Department of Hematology/Oncology

the Affiliated Children\'s Hospital of Nanjing Medical University

72 Guangzhou Rd，Nanjing,China

210008，+8618951769586

189邮箱

首页 联系人 附件中心 我的日程 收件箱 云会议 Re:Auth... Re:Auth... Re:Auth...

VIP fyj322@189.cn 升级VIP服务 手机版 设置 帮助 退出

点亮VIP图标

邮件搜索

写邮件

收件箱(4397)
星标邮件
已发送
草稿箱
垃圾箱
发票夹
微信文件
我的账单 (5)
其他文件夹 (694)
自定义标签
其他邮箱 (650)

返回 回复 回复全部 转发 删除 举报 标记为 移至 更多

Re: Authorship

发件人: 马晓朋 <maxiaopeng@njmu.edu.cn>

时 间: 2022年04月18日 10:39 星期一 展示更多

Agree Xiaopeng Ma

---- 回复的原邮件 ----

发件人	fyj322@189.cn
日期	2022年04月18日 09:33
收件人	王娅萍、maxiaopeng@njmu.edu.cn、15895323665@163.com<15895323665@163.com>、yunmeik_28@126.com、njchsxy@163.com、lhm13813743029@163.com、wyj15380168896@163.com、zhangheng0513@126.com、maomaozhu1993@163.com、yaoyao82986@126.com、jnjmu2021@163.com
主题	Authorship

Dear my colleagues,

Here we need to make an approvement regarding the authorship of manuscript entitled “Somatic FOXC1 insertion mutation remodels the immune microenvironment and promotes the progression of childhood acute lymphoblastic leukemia” which has been principle accepted by Cell Death & Disease (CDDIS-21-4509RRR). In detailed, Jie Huang was added as the co-first author of the article (ranking third). You, as a co-author of this article, agree to the above modifications and confirm that there is no conflict of interest. Please response whether you agree or not.

Thank you again for your work.

Best regards,

Yongjun fang M.D. Ph.D

Department of Hematology/Oncology

the Affiliated Children\'s Hospital of Nanjing Medical University

72 Guangzhou Rd，Nanjing,China

210008，+8618951769586

189邮箱
VIP fyj322@189.cn 升级VIP服务 手机版 设置 帮助 退出
点亮VIP图标
首页 联系人 附件中心 我的日程 收件箱 云会议 Re:Auth... Re:Auth...
邮件搜索
写邮件
收件箱(4398)
星标邮件
已发送
草稿箱
垃圾箱
发票夹
微信文件
我的账单 (5)
其他文件夹 (694)
自定义标签
其他邮箱 (650)
返回 回复 回复全部 转发 删除 举报 标记为 移至 更多

Re:Authorship

发件人： 孙晓燕 <njchsxy@163.com>

时 间： 2022年04月18日 10:34 星期一 展示更多

Agree+Xiaoyan Sun

--
发自我的网易邮箱手机智能版

在 2022-04-18 09:33:55，fyj322@189.cn 写道：

Dear my colleagues,

Here we need to make an approvement regarding the authorship of manuscript entitled “Somatic FOXC1 insertion mutation remodels the immune microenvironment and promotes the progression of childhood acute lymphoblastic leukemia” which has been principle accepted by Cell Death & Disease (CDDIS-21-4509RRR). In detailed, Jie Huang was added as the co-first author of the article (ranking third). You, as a co-author of this article, agree to the above modifications and confirm that there is no conflict of interest. Please response whether you agree or not.

Thank you again for your work.

Best regards,

Yongjun fang M.D. Ph.D

Department of Hematology/Oncology

the Affiliated Children\'s Hospital of Nanjing Medical University

72 Guangzhou Rd，Nanjing,China

210008，+8618951769586

Re: Authorship

发件人： 吴怡秀Y19310 <wyj15380168896@163.com>

时 间： 2022年04月18日 10:40 星期一 展示更多

Agree. Yijun Wu

---- Replied Message ----

From fyj322

Date 04/18/2022 09:33

To 王娅萍,
maxiaopeng@njmu.edu.cn,
15895323665@163.com<15895323665@163.com>,
yunmeik_28@126.com,
njchsxy@163.com,
lhm13813743029@163.com,
wyj15380168896@163.com,
zhangheng0513@126.com,
maomaozhu1993@163.com,
yaoyao82986@126.com,
jnjmu2021@163.com

Subject Authorship

Dear my colleagues,

Here we need to make an approvement regarding the authorship of manuscript entitled "Somatic FOXC1 insertion mutation remodels the immune microenvironment and promotes the progression of childhood acute lymphoblastic leukemia" which has been principle accepted by Cell Death & Disease (CDDIS-21-4509RRR). In detailed, Jie Huang was added as the co-first author of the article (ranking third). You, as a co-author of this article, agree to the above modifications and confirm that there is no conflict of interest. Please response whether you agree or not.

Thank you again for your work.

Best regards,

Yongjun fang M.D. Ph.D

Department of Hematology/Oncology

the Affiliated Children\'s Hospital of Nanjing Medical University

72 Guangzhou Rd，Nanjing,China

210008，+8618951769586

189邮箱
VIP fyj322@189.cn 升级VIP服务 手机版 设置 帮助 退出
首页 联系人 附件中心 我的日程 收件箱 云会议 Re:Auth... Re:Auth... Re:Auth... Re:Auth... Re:Auth... Re:Auth... Re:Auth... 回复：A... Re:Auth... 邮件搜索
写邮件
收件箱(4392)
星标邮件
已发送
草稿箱
垃圾箱
发票夹
微信文件
我的账单 (5)
其他文件夹 (694)
自定义标签
其他邮箱 (650)
返回 回复 回复全部 转发 删除 举报 标记为 移至 更多

Re: Authorship

发件人： YYY <yaoy1313@163.com>

时 间： 2022年04月19日 20:51 星期二 展示更多

Agree , Yue Yao

签名由 网易邮箱大师 定制

On 4/19/2022 20:51， wrote:

Dear my colleagues,

Here we need to make an approvement regarding the authorship of manuscript entitled "Somatic FOXC1 insertion mutation remodels the immune microenvironment and promotes the progression of childhood acute lymphoblastic leukemia" which has been principle accepted by Cell Death & Disease (CDDIS-21-4509RRR). In detailed, Jie Huang was added as the co-first author of the article (ranking third). You, as a co-author of this article, agree to the above modifications and confirm that there is no conflict of interest. Please response whether you agree or not.

Thank you again for your work.

Best regards,

Yongjun fang M.D. Ph.D

Department of Hematology/Oncology

the Affiliated Children\'s Hospital of Nanjing Medical University

72 Guangzhou Rd，Nanjing,China

210008，+8618951769586

快捷回复给:yaoy1313@163.com

189邮箱

fyj322@189.cn 升级VIP服务 手机版 设置 帮助 退出

点亮VIP图标

首页 联系人 附件中心 我的日程 收件箱 云会议 **Re:Auth...**

邮件搜索

写邮件

收件箱(4399)
星标邮件
已发送
草稿箱
垃圾箱
发票夹
微信文件
我的账单 (5)
其他文件夹 (694)
自定义标签
其他邮箱 (650)

返回 回复 回复全部 转发 删除 举报 标记为 移至 更多

Re: Authorship

发件人： 15895323665 <15895323665@163.com>

时 间： 2022年04月18日 10:30 星期一 展示更多

Agree. Xiaoyun Yang

---- Replied Message ----

From	fyj322
Date	04/18/2022 09:33
To	王娅萍,maxiaopeng@njmu.edu.cn,15895323665@163.com<15895323665@163.com>,yunmeik_28@126.com,njchsxy@163.com,lhm13813743029@163.com,wyj15380168896@163.com,zhangheng0513@126.com,maomaozhu1993@163.com,yaoyao82986@126.com,jnjmu2021@163.com
Subject	Authorship

Dear my colleagues,

Here we need to make an approvement regarding the authorship of manuscript entitled “Somatic FOXC1 insertion mutation remodels the immune microenvironment and promotes the progression of childhood acute lymphoblastic leukemia” which has been principle accepted by Cell Death & Disease (CDDIS-21-4509RRR). In detailed, Jie Huang was added as the co-first author of the article (ranking third). You, as a co-author of this article, agree to the above modifications and confirm that there is no conflict of interest. Please response whether you agree or not.

Thank you again for your work.

Best regards,

Yongjun fang M.D. Ph.D

Department of Hematology/Oncology

the Affiliated Children\'s Hospital of Nanjing Medical University

72 Guangzhou Rd，Nanjing,China

210008，+8618951769586

快捷回复给:15895323665@163.com

189邮箱
VIP fyj322@189.cn 升级VIP服务 手机版 设置 帮助 退出
首页 联系人 附件中心 我的日程 收件箱 云会议 Re:Auth... Re:Auth... Re:Auth... Re:Auth... Re:Auth... Re:Auth... Re:Auth... 回复：A... 邮件搜索
写邮件
收件箱(4392)
星标邮件
已发送
草稿箱
垃圾箱
发票夹
微信文件
我的账单 (5)
其他文件夹 (694)
自定义标签
其他邮箱 (650)
返回 回复 回复全部 转发 删除 举报 标记为 移至 更多

回复：Authorship

发件人： zhangheng0513@126.com <zhangheng0513@126.com>

时 间： 2022年04月18日 15:25 星期一 展示更多

Agree+ Heng Zhang

发自我的手机

-------- 原始邮件 --------

发件人： fyj322@189.cn

日期： 2022年4月18日周一 上午9:33

收件人： 王娅萍 , maxiaopeng@njmu.edu.cn, 15895323665@163.com, yunmeik_28@126.com, njchsxy@163.com, lhm13813743029@163.com, wyj15380168896@163.com, zhangheng0513@126.com, maomaozhu1993@163.com, yaoyao82986@126.com, jnjmu2021@163.com

主 题： Authorship

Dear my colleagues,

Here we need to make an approvement regarding the authorship of manuscript entitled “Somatic FOXC1 insertion mutation remodels the immune microenvironment and promotes the progression of childhood acute lymphoblastic leukemia” which has been principle accepted by Cell Death & Disease (CDDIS-21-4509RRR). In detailed, Jie Huang was added as the co-first author of the article (ranking third). You, as a co-author of this article, agree to the above modifications and confirm that there is no conflict of interest. Please response whether you agree or not.

Thank you again for your work.

Best regards,

Yongjun fang M.D. Ph.D

Department of Hematology/Oncology

the Affiliated Children\'s Hospital of Nanjing Medical University

72 Guangzhou Rd，Nanjing,China

210008，+8618951769586

189邮箱

fyj322@189.cn 升级VIP服务 手机版 设置 帮助 退出

首页 联系人 附件中心 我的日程 收件箱 云会议 Re:Auth... Re:Auth... Re:Auth... Re:Auth...

写邮件

收件箱(4396)
星标邮件
已发送
草稿箱
垃圾箱
发票夹
微信文件
我的账单 (5)
其他文件夹 (694)
自定义标签
其他邮箱 (650)

返回 回复 回复全部 转发 删除 举报 标记为 移至 更多

Re:Authorship

发件人：康美云 <yunmeik_28@126.com>

时 间：2022年04月18日 10:39 星期一 展示更多

Agree+Kang Meiyun

At 2022-04-18 09:33:55, fyj322@189.cn wrote:

Dear my colleagues,

Here we need to make an approvement regarding the authorship of manuscript entitled "Somatic FOXC1 insertion mutation remodels the immune microenvironment and promotes the progression of childhood acute lymphoblastic leukemia" which has been principle accepted by Cell Death & Disease (CDDIS-21-4509RRR). In detailed, Jie Huang was added as the co-first author of the article (ranking third). You, as a co-author of this article, agree to the above modifications and confirm that there is no conflict of interest. Please response whether you agree or not.

Thank you again for your work.

Best regards,

Yongjun fang M.D. Ph.D

Department of Hematology/Oncology

the Affiliated Children\'s Hospital of Nanjing Medical University

72 Guangzhou Rd，Nanjing,China

210008，+8618951769586

快捷回复给:yunmeik_28@126.com
