# Supplementary material for: Somatic FOXC1 insertion mutation remodels the immune microenvironment and promotes the progression of childhood acute lymphoblastic leukemia
Source: Cell Death Dis. 2022 May 3;13(5):431. doi: 10.1038/s41419-022-04873-y (PMC9065155; doi:10.1038/s41419-022-04873-y)
Supplement: Supplementary file 3 — SUPPLEMENTAL MATERIAL [file 41419_2022_4873_MOESM3_ESM.pdf]

Supplementary Tables

Supplementary Table 1. Primer information

Gene name		Sequence
FOXC1	Forward	AACCTGCAAGCCATGAGCC
	Reverse	ACCGAGTGGAAGTTCTGCTG
GAPDH	Forward	GGAGCGAGATCCCTCCAAAAT
	Reverse	GGCTGTTGTCATACTTCTCATGG
KLF10	Forward	CTTCCGGGAACACCTGATTTT
	Reverse	GCAATGTGAGGTTTGGCAGTATC
KLF1	Forward	GGTTGCGGCAAGAGCTACA
	Reverse	GTCAGAGCGCGAAAAAGCAC
KLF2	Forward	TTCGGTCTCTTCGACGACG
	Reverse	TGCGAACTCTTGGTGTAGGTC
KLF4	Forward	CCCACATGAAGCGACTTCCC
	Reverse	CAGGTCCAGGAGATCGTTGAA
KLF5	Forward	CCTGGTCCAGACAAGATGTGA
	Reverse	GAACTGGTCTACGACTGAGGC
KLF13	Forward	CGGCCTCAGACAAAGGGTC
	Forward	TTCCCGTAAACTTTCTCGCAG
BSP FOXC1	Reverse	TTTAAAGTAGGAAAAGTTAAAGGAAT
	Forward	AAAATAACACTATCCTTTAATAAACCAAAA

Supplementary Table 2. Summary of shRNA Oligos

Name	Oligo Sequence
KLF10 shRNA-1	CACCGCGCGATTATGCAATTATATTCGAAAATAT<br>AAAAGCGCGATTATGCAATTATATTTTCGAATATAA<br>TTGCATAATCGCGC
KLF10 shRNA-2	CACCGCACTGGTTTCAAGAATATGGCGAACCATAT<br>TCTTGAAACCAGTGC<br>AAAAGCACTGGTTTCAAGAATATGGTTCGCCATAT<br>TCTTGAAACCAGTGC
KLF10 shRNA-3	CACCGCTAAATGACATTGCTCTACCCGAAGGTAGA<br>GCAATGTCATTTAGC<br>AAAAGCTAAATGACATTGCTCTACCTTCGGGTAGA<br>GCAATGTCATTTAGC

Supplementary Table 3. Detailed information of candidate mutations

Chr	Position	Gene Symbol	Ref	Alt	Amino acid alter
chr15	23086364	NIPA1	GGCC	G	AA15A
chr17	48227384	PPP1R9B	G	GGC	A164G?
chr18	21124945	NPC1	C	G	M642I
chr3	40503520	RPL14	A	ACTGCTG	T149TAA
chr4	140811063	MAML3	TTGCTGCTGCTGC	T	QQ505
chr6	1612017	FOXC1	A	ACGG	H446HG
chr6	4492840	HLA-DPB1	G	A	V105I
chr8	103573010	ODF1	CTGCAACCCCTGCA GCCCCTGCAACCCG	C	CNPCSPCNP218
chr8	77765298	ZFHX4	ACCT	A	P2048
chr10	76788689	KAT6B	GGAA	G	E1370

Chr: Chromosome

Ref: Reference sequence

Alt: Altered sequence

Supplementary Table S4. Summary of antibodies

Name	Catalog#	Company
Anti-CD4	ab213215	Abcam
Anti-CD8	ab217344	Abcam
Anti-CD3	ab16669	Abcam
Anti-CD25	ab283576	Abcam
Anti-Foxp3	ab36607	Abcam
Anti-IFN-γ	ab224197	Abcam

Supplementary Figure legends

Supplementary Figure 1. Construction of mutant type FOXC1. a: The Schematic diagram of GeneCopoeia's IndelCheck™ CRISPR insertion or deletion (indel) detection system. b: The PCR assay confirmed the mutant and wild type of FOXC1. C: Sanger sequencing for the PCR products.

Supplementary Figure 2. Distribution of T helper cells. a: The percentage of Th1 cells. b: The percentage of Th2 cells. c: The percentage of Th17 cells. n. s. indicated no significance.

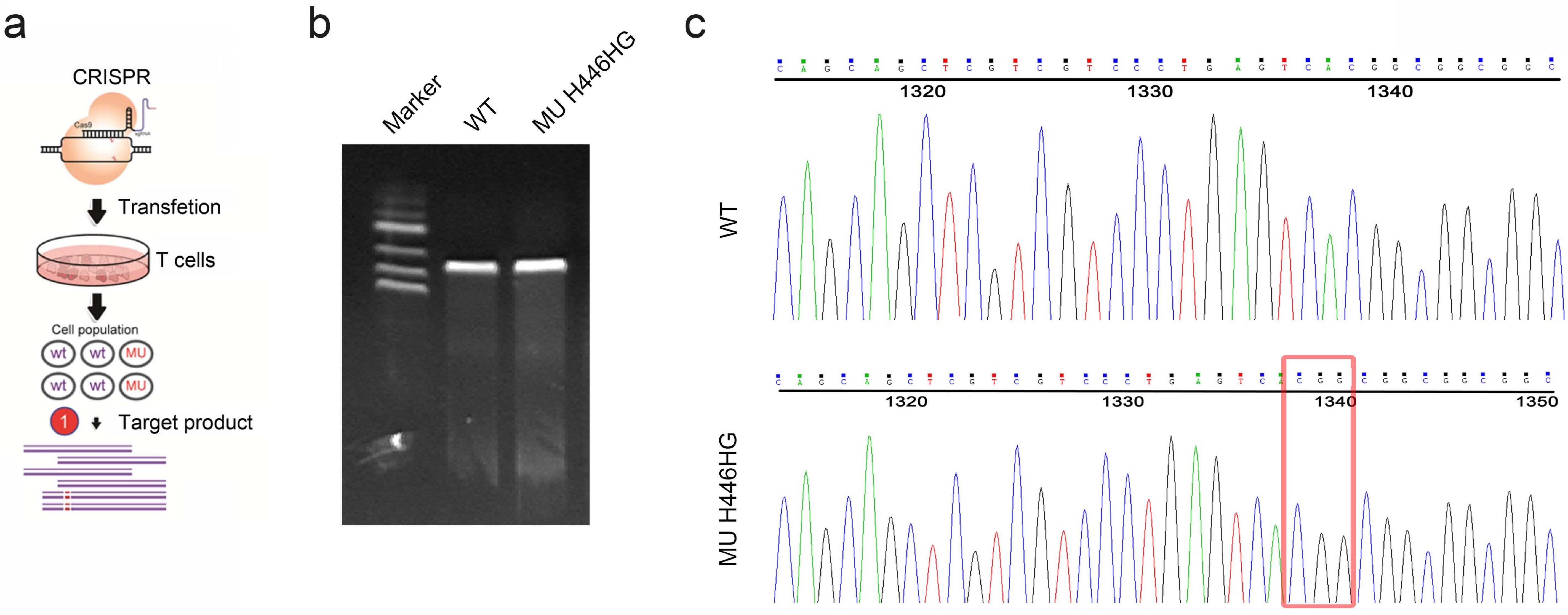

a
CRISPR
Cas9
Transfetion
T cells
Cell population
wt
MU
1
Target product
b
Marker
WT
MU H446HG
c
1320
1330
1340
1350
WT
MU H446HG

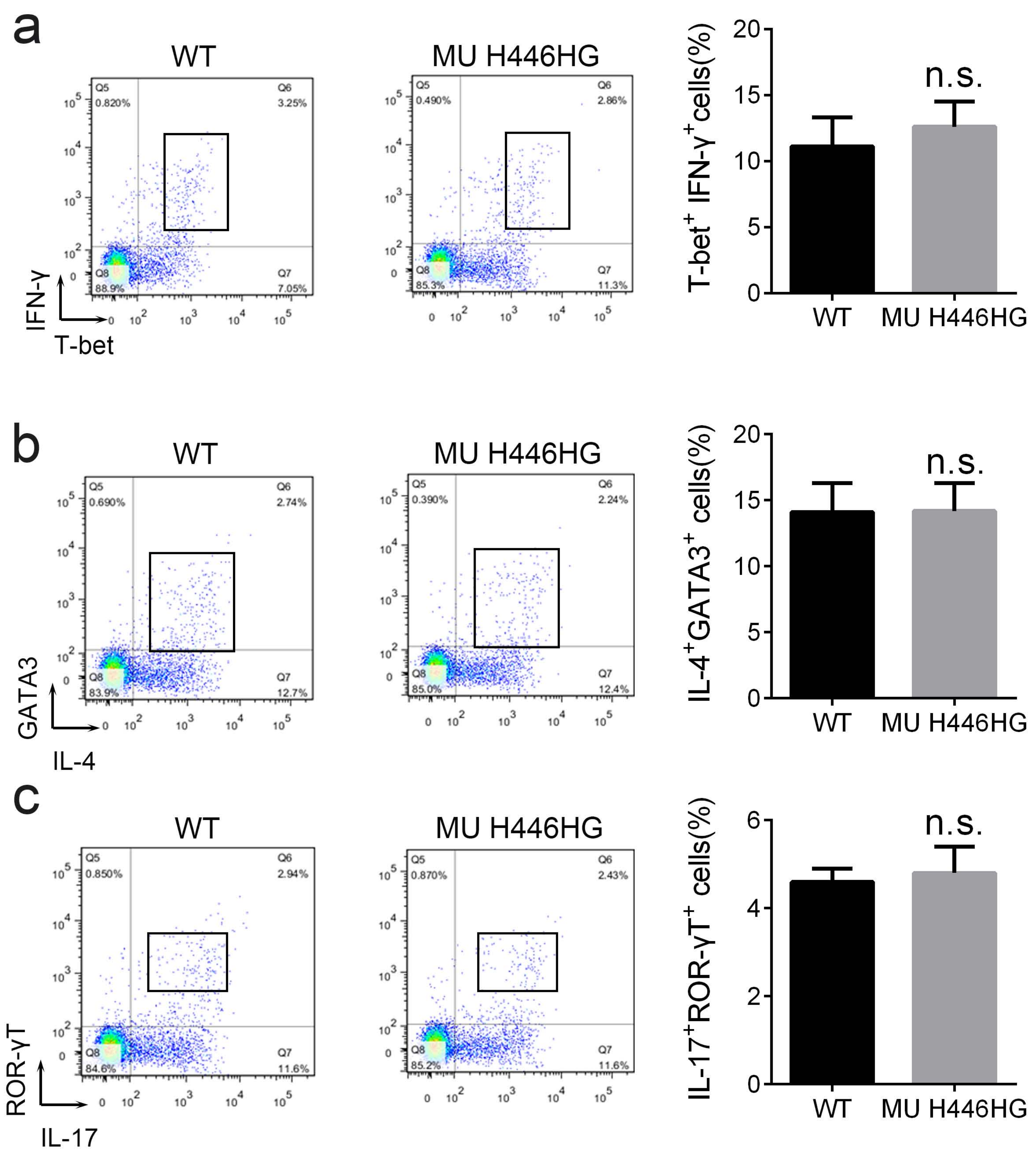

a
WT
MU H446HG
Q5 0.820%
Q6 3.25%
Q8 88.9%
Q7 7.05%
Q5 0.490%
Q6 2.86%
Q8 85.3%
Q7 11.3%
IFN-γ
T-bet
T-bet+ IFN-γ+cells(%)
n.s.
WT
MU H446HG
b
WT
MU H446HG
Q5 0.690%
Q6 2.74%
Q8 83.9%
Q7 12.7%
Q5 0.390%
Q6 2.24%
Q8 85.0%
Q7 12.4%
GATA3
IL-4
IL-4+GATA3+ cells(%)
n.s.
WT
MU H446HG
c
WT
MU H446HG
Q5 0.850%
Q6 2.94%
Q8 84.6%
Q7 11.6%
Q5 0.870%
Q6 2.43%
Q8 85.2%
Q7 11.6%
ROR-γT
IL-17
IL-17+ROR-γT+ cells(%)
n.s.
WT
MU H446HG

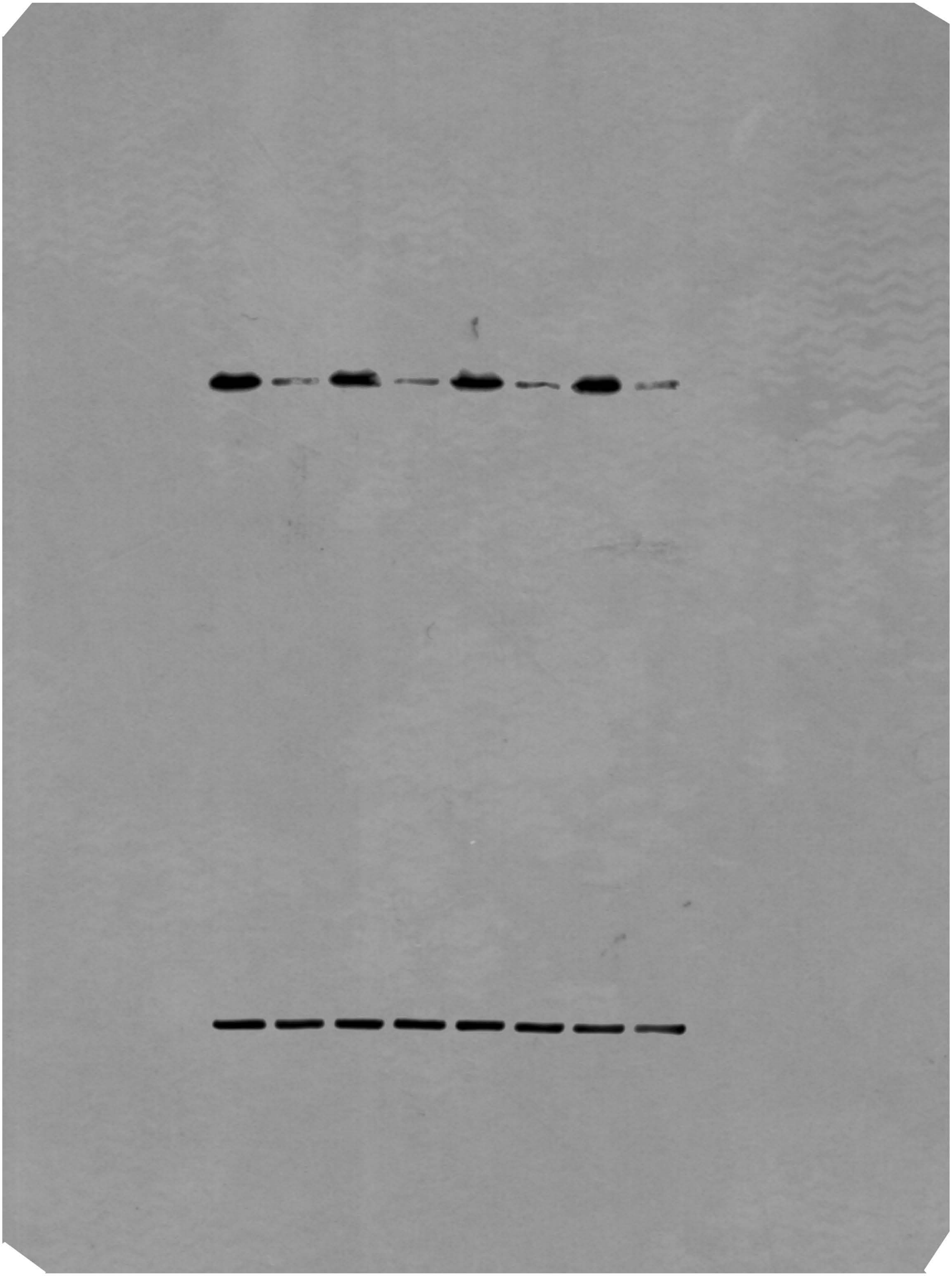

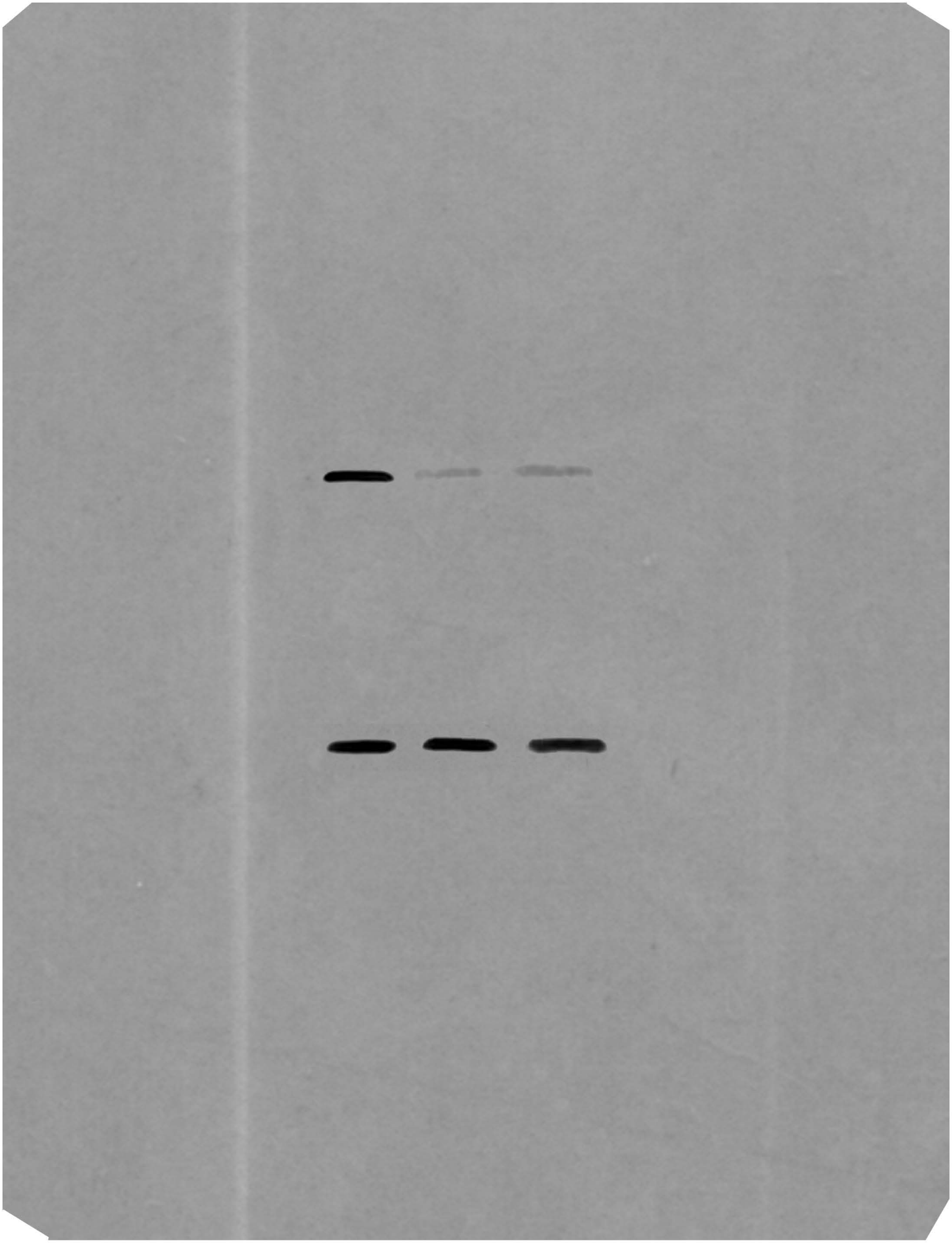

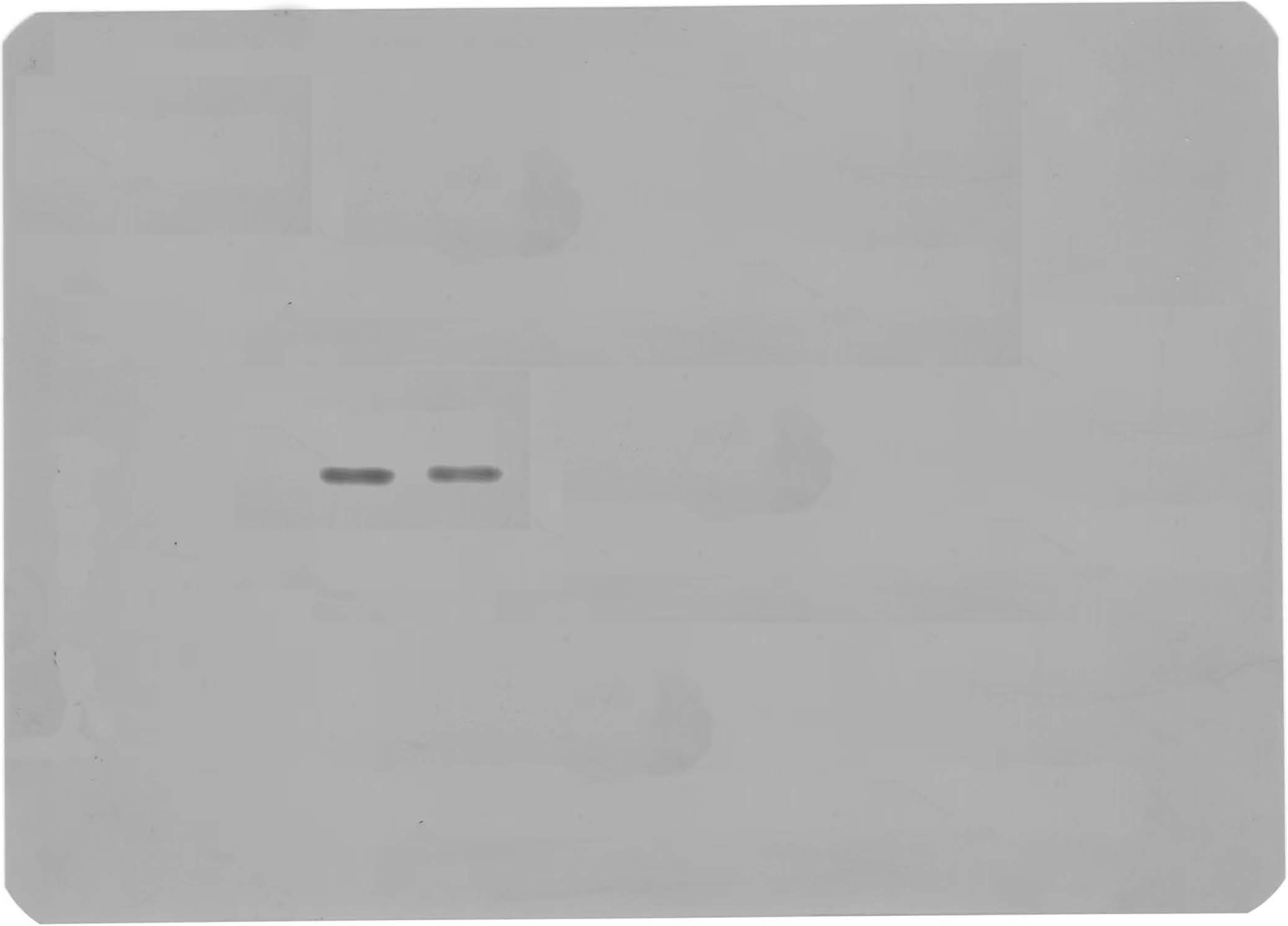

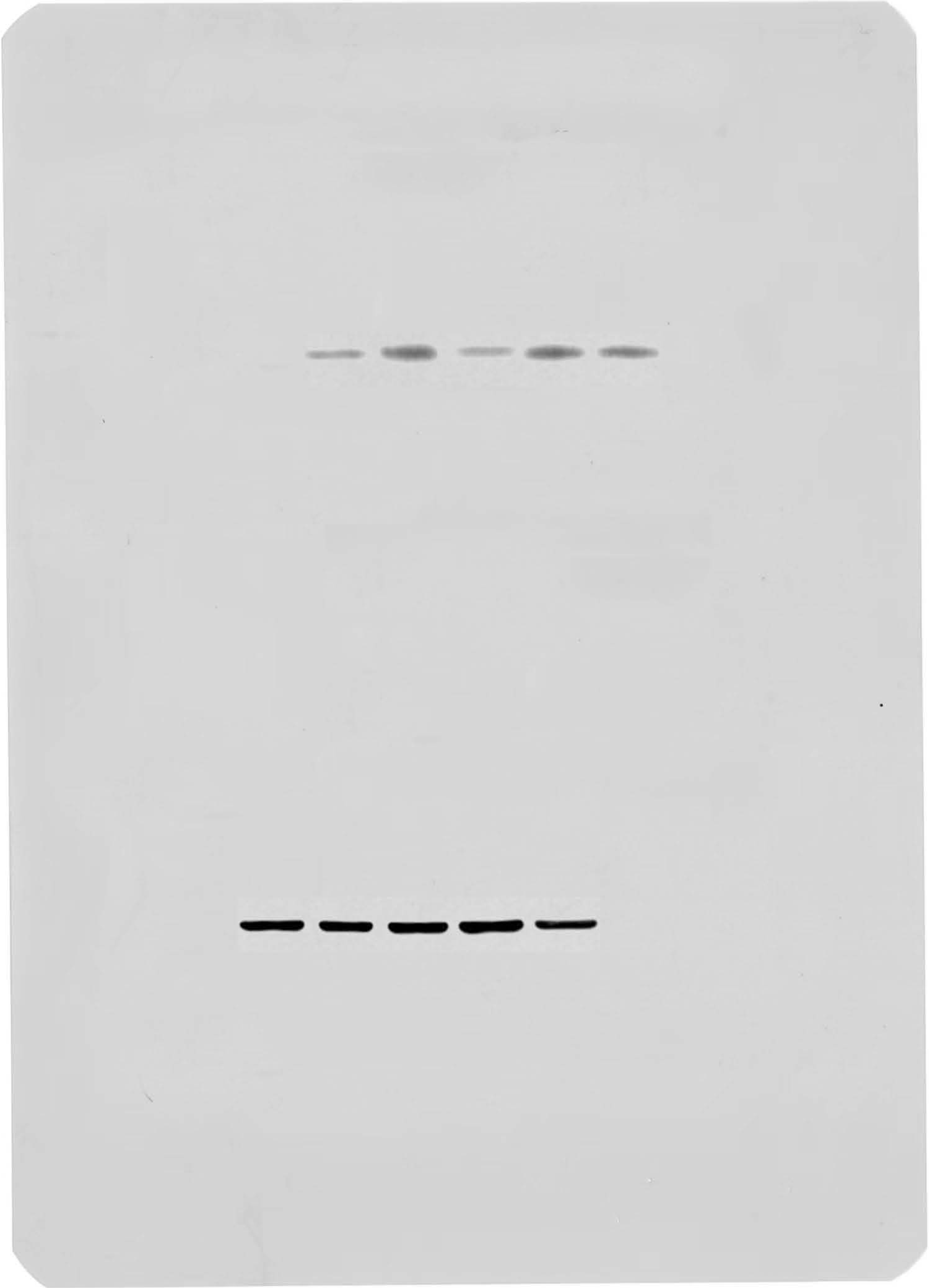

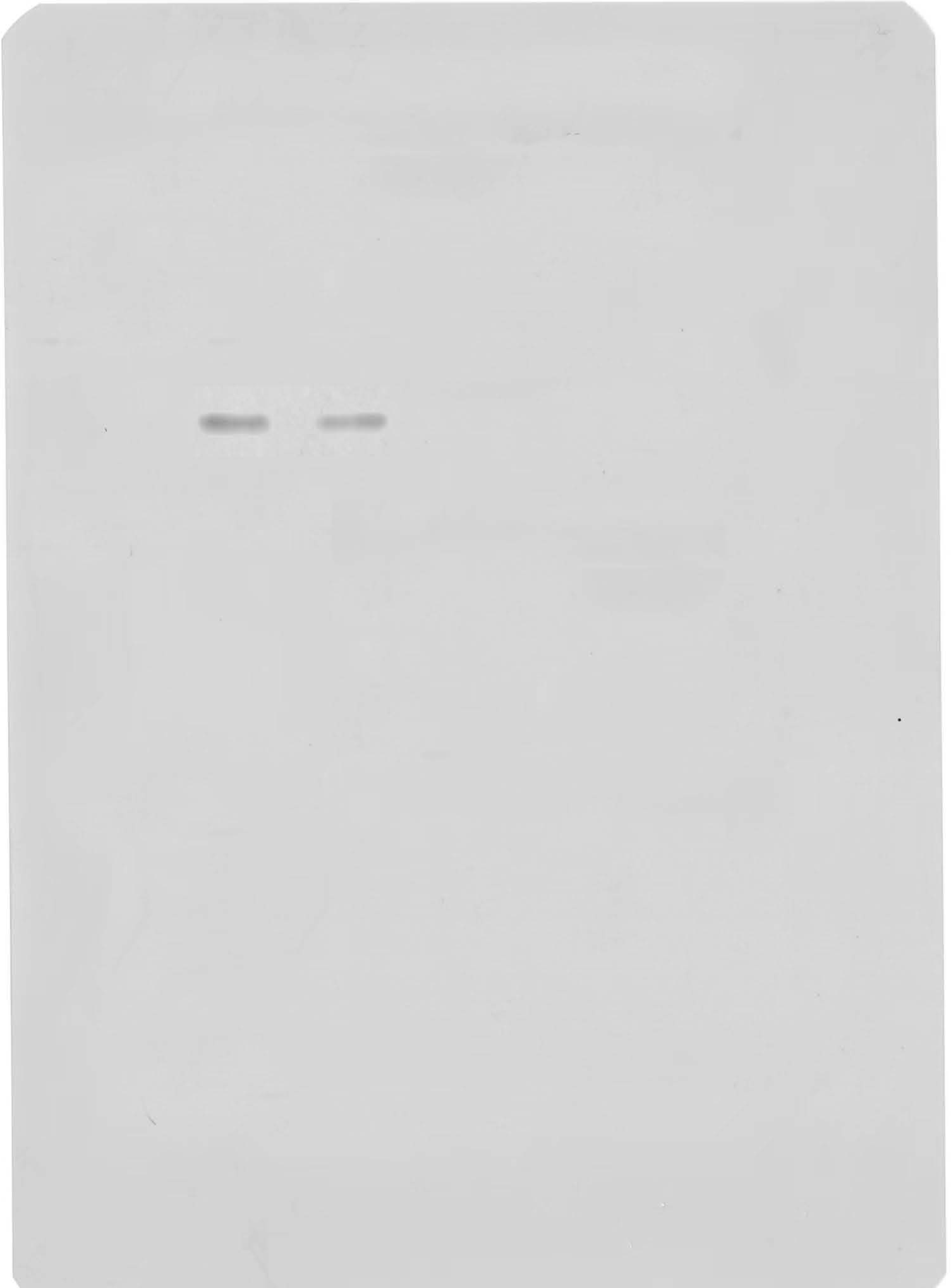

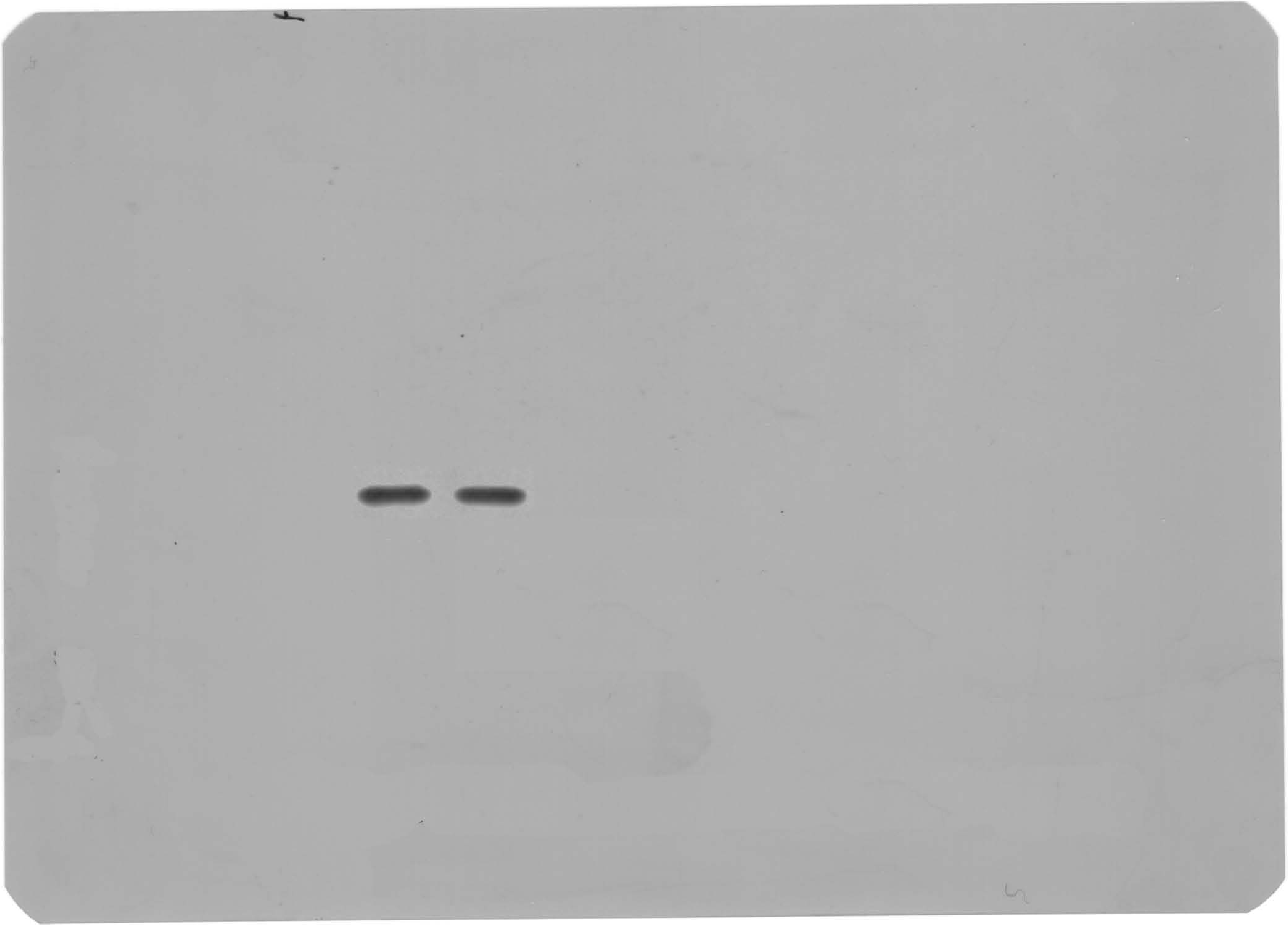

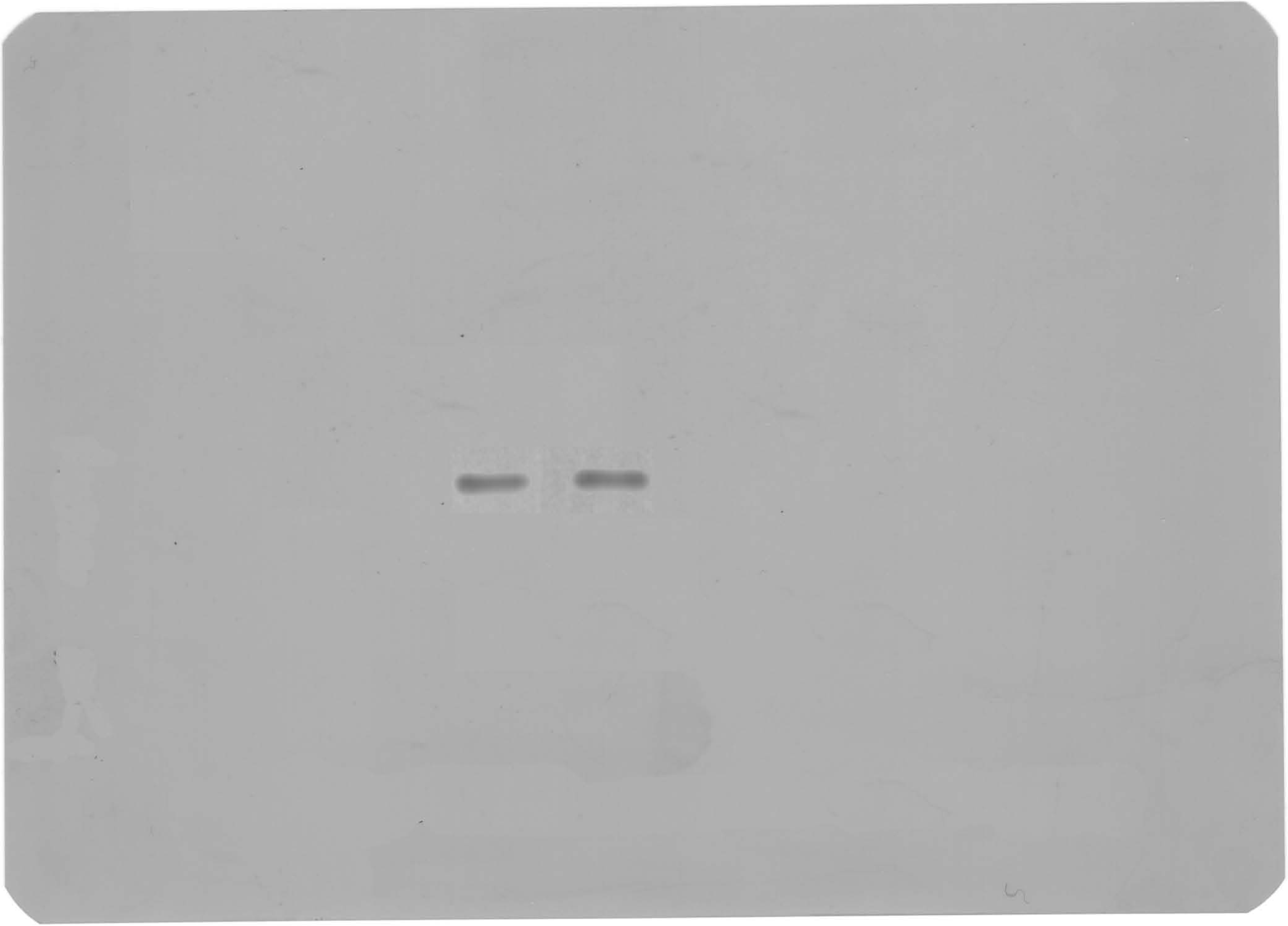

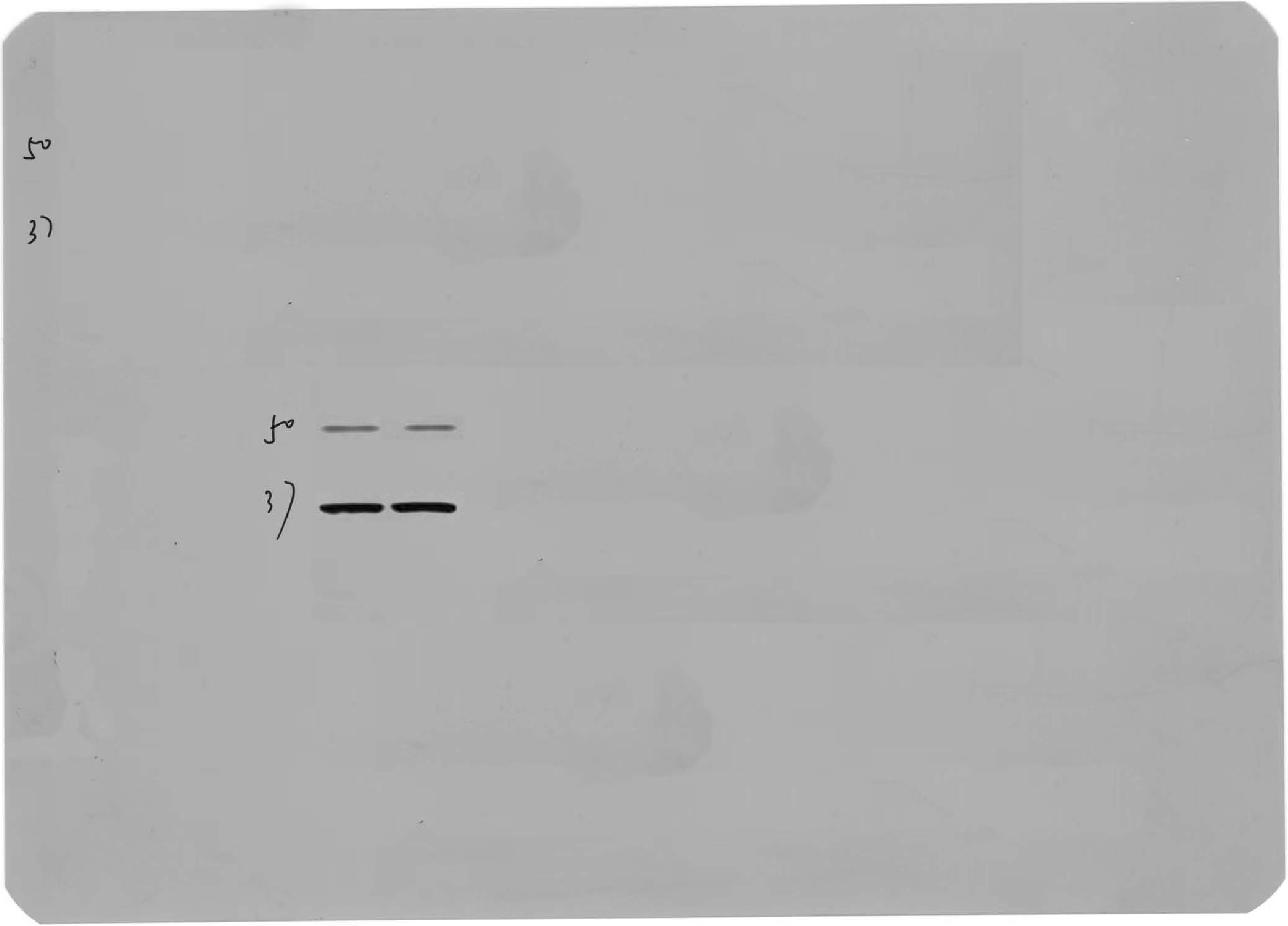
50
37
50
37
